# Impaired NHEJ repair in amyotrophic lateral sclerosis is associated with TDP-43 mutations

**DOI:** 10.1186/s13024-020-00386-4

**Published:** 2020-09-09

**Authors:** Anna Konopka, Donna R. Whelan, Md Shafi Jamali, Emma Perri, Hamideh Shahheydari, Reka P. Toth, Sonam Parakh, Tina Robinson, Alison Cheong, Prachi Mehta, Marta Vidal, Audrey M. G. Ragagnin, Ivan Khizhnyak, Cyril J. Jagaraj, Jasmin Galper, Natalie Grima, Anand Deva, Sina Shadfar, Garth A. Nicholson, Shu Yang, Suzanne M. Cutts, Zuzana Horejsi, Toby D. M. Bell, Adam K. Walker, Ian P. Blair, Julie D. Atkin

**Affiliations:** 1 Centre for MND Research, Department of Biomedical Sciences, Faculty of Medicine & Health Sciences, Macquarie University, 75 Talavera Road NSW, North Ryde, NSW 2109 Australia; 2 Department of Pharmacy and Biomedical Sciences, La Trobe Institute for Molecular Science, La Trobe University, Bendigo, VIC Australia; 3 Department of Biochemistry and Genetics, La Trobe Institute for Molecular Science, Bundoora, VIC Australia; 4 Brain and Mind Centre, Central Clinical School, Faculty of Medicine and Health, University of Sydney, Camperdown, NSW Australia; 5 Department of Plastic and Reconstructive Surgery, Macquarie University, and The Integrated Specialist Healthcare Education and Research Foundation, Sydney, Australia; 6 ANZAC Research Institute, Concord Hospital, University of Sydney, Sydney, NSW Australia; 7 Barts Cancer Institute, Queen Mary University of London, London, UK; 8 School of Chemistry, Monash University, Wellington Road, Clayton, VIC Australia; 9 Neurodegeneration Pathobiology Laboratory, Queensland Brain Institute, The University of Queensland, St Lucia, Queensland Australia

**Keywords:** DNA damage, TDP-43 mutations, NHEJ, Super-resolution microscopy

## Abstract

**Background:**

Pathological forms of TAR DNA-binding protein 43 (TDP-43) are present in motor neurons of almost all amyotrophic lateral sclerosis (ALS) patients, and mutations in TDP-43 are also present in ALS. Loss and gain of TDP-43 functions are implicated in pathogenesis, but the mechanisms are unclear. While the RNA functions of TDP-43 have been widely investigated, its DNA binding roles remain unclear. However, recent studies have implicated a role for TDP-43 in the DNA damage response.

**Methods:**

We used NSC-34 motor neuron-like cells and primary cortical neurons expressing wildtype TDP-43 or TDP-43 ALS associated mutants (A315T, Q331K), in which DNA damage was induced by etoposide or H_2_O_2_ treatment. We investigated the consequences of depletion of TDP-43 on DNA repair using small interfering RNAs. Specific non homologous end joining (NHEJ) reporters (EJ5GFP and EJ2GFP) and cells lacking DNA-dependent serine/threonine protein kinase (DNA-PK) were used to investigate the role of TDP-43 in DNA repair. To investigate the recruitment of TDP-43 to sites of DNA damage we used single molecule super-resolution microscopy and a co-immunoprecipitation assay. We also investigated DNA damage in an ALS transgenic mouse model, in which TDP-43 accumulates pathologically in the cytoplasm. We also examined fibroblasts derived from ALS patients bearing the TDP-43 M337V mutation for evidence of DNA damage.

**Results:**

We demonstrate that wildtype TDP-43 is recruited to sites of DNA damage where it participates in classical NHEJ DNA repair. However, ALS-associated TDP-43 mutants lose this activity, which induces DNA damage. Furthermore, DNA damage is present in mice displaying TDP-43 pathology, implying an active role in neurodegeneration. Additionally, DNA damage triggers features typical of TDP-43 pathology; cytoplasmic mis-localisation and stress granule formation. Similarly, inhibition of NHEJ induces TDP-43 mis-localisation to the cytoplasm.

**Conclusions:**

This study reveals that TDP-43 functions in DNA repair, but loss of this function triggers DNA damage and is associated with key pathological features of ALS.

## Background

Amyotrophic lateral sclerosis (ALS) is a rapidly progressing neurodegenerative disorder associated with atrophy of both upper and/or lower motor neurons, leading to muscle wasting due to denervation [1]. ALS overlaps clinically and pathologically with frontotemporal dementia (FTD) [2], which manifests as neuronal degeneration in the frontal and temporal lobes, causing progressive deterioration of language, personality, and behaviour [3]. Pathological forms of TAR DNA-binding protein 43 (TDP-43), a heterogenous nuclear ribonucleoprotein (hnRNP), are present in affected motor neurons in sporadic disease and almost all ALS cases (97%) [4, 5]. In this form of disease, TDP-43 aberrantly accumulates in the cytoplasm where it is recruited to newly formed stress granules (SGs), which are linked to abnormal protein aggregation and pathology in ALS [6, 7]. Furthermore, recent studies have highlighted the importance of SGs in the pathogenesis of ALS. Optogenetic induction of SGs is cytotoxic and results in TDP-43 pathology [8], whereas preventing recruitment of TDP-43 to SGs in induced pluripotent stem cells (iPSCs) prevents TDP-43 accumulation [9]. Similarly, TDP-43 pathology is present in a significant proportion of FTD cases (45%, [4]) and pathological forms of TDP-43 induce toxicity and neurodegeneration [10, 11]. Importantly, inhibition of TDP-43 pathology and restoration of nuclear TDP-43 leads to neuronal preservation, muscle re-innervation and functional recovery in mouse models [12]. Together these studies highlight a prominent and central role for pathological forms of TDP-43 in both ALS and FTD. However, it remains unclear how TDP-43 pathology is induced in ALS or how this is related to neurodegeneration.

TDP-43 is a RNA- and DNA-binding protein normally located in the nucleus, where it is involved in transcription, splicing, RNA metabolism and miRNA biogenesis [13]. Whilst the roles of TDP-43 in RNA metabolism have been well-studied, its role in DNA-related processes remain relatively undefined. Mutations in TDP-43, including A315T, Q331K, and M337V, induce TDP-43 pathology and are present in 4–5% of familial ALS [14]. The pathogenic mechanisms associated with TDP-43 are thought to involve both loss of nuclear function and coincident gain of toxic function in the cytoplasm [15].

The genome is under constant attack from both environmental agents and normal metabolic processes. Thus, maintaining genomic integrity is essential because damage to DNA can induce mutations and seriously compromise cellular viability. DNA damage induces the ‘DNA damage response (DDR)’, signalling pathways that aim to detect and repair the damage. However, persistent DNA damage and defects in DNA repair lead to apoptosis. The most cytotoxic lesions are double-stranded DNA breaks (DSBs) which are primarily repaired by error-prone non-homologous end joining (NHEJ) in neurons [16], unlike in other cells where homologous recombination (HR) is also present. There are two mechanistically distinct NHEJ pathways; classical NHEJ, which leads to minimal sequence alternations at the repair site, and alternative NHEJ, which causes extensive deletions and/or insertions in these regions [17]. The catalytic subunit of DNA-dependent protein kinase (DNA-PKcs) is a core component of the NHEJ machinery that forms foci during repair [18]. The lack of HR in neurons means there are fewer options to repair DNA compared to other cell types. Furthermore, whilst most other cells are continuously replaced and hence can tolerate the loss of any cells harbouring irreparable DNA lesions through apoptosis, neurons are post-mitotic and therefore subjected to a lifetime of damage [19]. Hence, neurons are particularly susceptible to DNA damage. In addition, neurons are vulnerable to oxidative stress, which induces DNA damage primarily by single stranded breaks (SSBs), but they readily convert to DSBs [20].

Fused in Sarcoma (FUS) has striking functional and pathological similarities to TDP-43 in ALS, and it normally performs important functions in DNA repair [21]. Interestingly, mutations in genes encoding DDR proteins are increasingly associated with ALS, including *SETX, VCP, NEK1, C21orf2,* and *CCNF* [22–27]. Recently we demonstrated that mutations in *C9ORF72*, which cause a major proportion of familial ALS cases, induce DNA damage in neuronal cells and motor neurons of ALS patients [28]. This was subsequently linked to deficiencies in R loop repair and defective ubiquitylation of H2A, which impair DSB signalling [29]. TDP-43 has also been implicated in R-loop repair [30] and two recent studies link TDP-43 to DNA damage [31, 32]. It has also been reported that TDP-43 is a component of NHEJ, acting as a scaffold for recruitment of the break-sealing XRCC4-DNA ligase 4 complex. Moreover, nuclear loss of TDP-43 is linked to defects in the repair of DSBs [31], and expression of TDP-43 mutant Q331K perturbs repair of DSBs, by inhibiting NHEJ associated DNA Ligase 4 [32]. These studies imply that DNA damage is important in the pathogenesis of ALS.

In this study we demonstrate that TDP-43 is a DNA repair protein that functions in classical, but not alternative, NHEJ in neuronal cells. However, ALS TDP-43 mutants A315T and Q331K lack this normally protective function, leading to the accumulation of DNA damage. Furthermore, in mouse models displaying pathological TDP-43 that develop features of ALS (brain atrophy, muscle denervation, motor neuron loss, and motor impairment leading to death), DNA damage is present before the onset of disease. These mice express cytoplasmic, wildtype TDP-43 and thus bear semblance to sporadic ALS. We also show that DNA damage induces features typical of TDP-43 pathology: mis-localisation to the cytoplasm and SG formation. Moreover, inhibition of classical NHEJ repair also leads to TDP-43 mis-localisation to the cytoplasm. This study therefore places aberrant DNA damage responses onto the pathophysiolology of TDP-43, which is associated with almost all ALS cases. It therefore highlights the DDR as a novel therapeutic target for ameliorating TDP-43 pathology in ALS.

## Methods

### Human samples

Details of the patients used in this study are summarised in Table 1:

**Table 1:** Dermal fibroblasts

Case	Mutation status	Age at collection (Year)	Sex
Control 1 (without any significant pathology)	NA	36	Male
Control 2 (without any significant pathology)	NA	65	Female
Control 3 (without any significant pathology)	NA	34	Female
TDP-43 presymptomatic 1	TDP-43 M337V	46	Female
TDP-43 presymptomatic 2	TDP-43 M337V	42	Female
TDP-43 affected/ALS	TDP-43 M337V	45	Male

### Animals

Monogenic B6;C3-Tg (NEFH-tTA)8Vle/J (NEFH-tTA line 8, stock #025397) mice and monogenic B6;C3-Tg (tetO-TARDBP*)4Vle/J (tetO-hTDP-43ΔNLS line 4, stock #014650) mice were obtained from the Jackson Laboratory (USA) and maintained on a mixed B6/C3H background (produced from F1 offspring of C57BL/6JArc and C3H/HeJArc mice, Animal Resources Centre, Australia) and were crossed to obtain bigenic rNLS and littermate control mice. All the mice were genotyped according to the Jackson Laboratory protocols. Expression of the hTDP-43–rNLS transgene was confirmed by cytoplasmic TDP-43 staining and increased levels of human TDP-43 by immunoblotting. Mice were provided with feed containing 200 mg/kg doxycycline (Gordon Specialty Feeds) until 6–8 weeks of age.

### Human fibroblast cultures

Fibroblasts from both ALS patients and pre-symptomatic carriers bearing the M337V mutation were obtained and cultured as previously described [33]. Control fibroblasts were derived from superficial skin biopsies from neurologically normal control individuals recruited from the plastic and reconstructive surgery clinic, Macquarie University, according to informed written consent. Briefly, fibroblasts from the biopsy specimens were cultured in Dulbecco’s modified Eagle’s medium (Sigma Aldrich) supplemented with 2 mM L-glutamine, 100 U/ml penicillin, 100 mg/ml streptomycin and 10% heat-inactivated foetal bovine serum (Sigma Aldrich). Cells were maintained in a humidified 37 °C incubator with 5% CO2 and used for experiments at passage number 5 or 6.

### Growth of primary cortical neurons

Primary cortical neuronal cultures were prepared from mouse C57BL/6 E16 brains. After isolation, cells were plated on glass coverslips covered with poly-L-lysine (Sigma-Aldrich), in B27 neurobasal medium supplemented with 1% glutamate and 1% penicillin/streptomycin and incubated at 37 °C and 5% CO_2_. Neurons were transfected with Lipofectamine 2000 at 3 or 5 DIV and fixed with 4% paraformaldehyde after 24 h.

### Cell lines

NSC-34/CHO-9/XR-C1 or Neuro2A cells were cultured in DMEM medium with 10% FBS at 37 °C and 5% CO_2._

### Cells treatment

DNA damage was induced by treatment with 13.5 μM etoposide or 100 μM H_2_O_2_ for 1 h. For inhibition of DNA-PK activity, a specific inhibitor was used, NU 7441 (Tocris, 20 nM).

### Immunocytochemistry

After washing with 1 x PBS, cells were permeabilised in 0.1% Triton X-100 in PBS for 5 min and blocked in 1% BSA in PBS for 30 min. Staining was performed overnight with the following primary antibodies at a concentration of 1:500: rabbit anti-γH2AX (Novus Biologicals); rabbit anti-53BP1 (Novus Biologicals), mouse anti-DNA-PKcs (Abcam), rabbit anti-TDP-43 C-terminus (405–414) (Cosmo Bio Co., LTD), and rabbit anti-phospho-TDP-43 (Cosmo Bio Co., LTD). The following secondary antibodies were used: anti-rabbit Alexa Fluor 488, (Life technologies), anti-mouse Alexa Fluor 594 (Life technologies) or anti-rabbit Alexa Fluor 647 antibodies (all Life Technologies) for 2 h. The nuclei were counterstained with Hoechst 33342 (Sigma-Aldrich) or DAPI (Sigma Aldrich).

### Single cell gel electrophoresis assay (Comet assay)

The Comet assay was adapted from a previous method [34]. Briefly, cells were mixed with 1% Type VII molten agarose and pipetted onto the precoated slides. Cells were lysed in lysis buffer (100 mM Na2EDTA, 10 mM Tris, 2.5 M NaCl, pH10.5) containing 1% Triton-100 and incubated on ice for 1 h in the dark. After washing, the slides were transferred to an electrophoresis tank. Electrophoresis was performed in the dark for 30 min at 30 V. After electrophoresis, cells were fixed and stained with SYBR Safe. The images were taken on an Olympus BX40 microscope and analysed with Komet 5.5 software by Kinetic Imaging. The analysis was performed blind.

### siRNA TDP-43 knockdown

Two small interfering RNAs (siRNAs) described previously [35], targeting mouse TDP-43 or non-targeting scrambled control, were transfected into NSC-34 cells with Lipofectamine 2000 (Invitrogen). After 72 h transfection, cells were treated with 13.5 μM etoposide for 1 h and fixed for immunocytochemistry or lysed for immunoblotting.

### Immunohistochemistry

After deparaffinisation, antigen retrieval in 10 mM citrate buffer with 0.1% Tween for 30 min at 80 °C was performed. Sections were blocked with normal donkey serum for 30 min and then stained with anti-γH2AX (1:200, Novus Biologicals) or anti-human TDP-43 antibodies (1:1000, Proteintech) for 24 h at 4 °C, and after three washes with PBS, the tissues were incubated with AlexaFluor 488/564 (1:200, Molecular Probes) conjugated secondary antibodies for 2 h at 4 °C. The nuclei were counterstained with Hoechst 33342 (Sigma-Aldrich).

### Image acquisition

Fluorescently labelled cells were acquired using a Zeiss AxioImager epi-fluorescence microscope (Zeiss) or a Zeiss LSM 880 confocal microscope.

### Plasmids

The following mammalian expression plasmids were used as described previously: pimEJ5GFP (Addgene # 44026) and EJ2GFP-puro (Addgene # 44025) were a gift from Jeremy Stark, TDP-43 wildtype-EGFP, TDP-43 A315T-EGFP, TDP-43 Q331K-EGFP, pEGFP-N1 [36], TDP-43 wildtype-mCherry, TDP-43 A315T-mCherry, TDP-43 Q331K-mCherry, empty pmCherry-N1 [37].

### Western blotting

Mouse brain tissues were thawed on ice and sonicated in 5x volumes of BICARB buffer (100 mM ammonium bicarbonate, 1% sodium deoxycholate, pH 7.6). Samples were centrifuged at 100,000 x g at 4 °C for 30 min. Supernatants were collected as BICARB soluble fractions. The human fibroblasts or NSC-34 cells were lysed in 50 mM Tris-HCl (pH 7.4), 150 mM NaCl, 0.1% (wt/v) SDS, 1% protease inhibitor cocktail (Sigma) and 1% phosphatase inhibitor cocktail (Sigma). Protein concentrations were determined using a BCA protein assay (Pierce). Twenty micrograms of soluble fractions were analysed by 4–15% gradient SDS-PAGE and blotted onto nitrocellulose membranes (Bio-Rad). Blots were blocked with 5% skim milk and incubated with primary antibodies diluted in blocking buffer overnight at 4 °C. The next day, unbound primary antibodies were removed by washing and blots were incubated with HRP-conjugated secondary antibodies for 1–2 h at room temperature. Images were developed with ECL blotting substrates (Bio-Rad) and a ChemiDoc Gel imaging system (Bio-Rad). Antibodies used for immunoblotting were: rabbit anti-γH2AX (1:1000, NB-100-384, Novus Biological), mouse anti-GAPDH (1:4000, 60,004-Ig, Proteintech), rabbit anti-GAPDH (1:4000, G8795, Merck), rabbit anti-TDP-43 (1:1000, TIP-TD-P09, Cosmo Bio Co.), goat anti-mouse IgG and IgM HRP conjugate (1:4000, Ap130P, Merck EMD Millipore), and goat anti-rabbit IgG antibodies, peroxidase conjugated (1:4000, Ap132P, Merck EMD Millipore).

### Immunoprecipitation

Immunoprecipitation was performed using GFP-Trap MA (Chromotek), which utilizes small recombinant alpaca antibody fragments covalently coupled to the surface of agarose magnetic beads. Briefly, NSC-34 transfected cells were lysed in lysis buffer (20mM Tris-HCl, 150mM NaCl, 2mM EDTA, 1% NP40 pH 7.4) with a cocktail of protease inhibitors (Roche) and 1 m PMSF for 30 min. on ice. Then, cell lysates were centrifuged at 20,000 x g for 10 min at 4 °C. After centrifugation, the lysates were transferred to a pre-cooled new tube. The protein concentration was quantified using a BCA kit (Thermo Fisher Scientific). Five hundred microgram of the lysate was taken for further processing. Fifty microliter of diluted lysates were retained for immunoblotting analysis for the input samples. Twenty five microliter of equilibrated GFP-Trap MA beads were added to diluted lysates and rotated end-over-end overnight at 4 °C. The following day, the beads were separated magnetically from the supernatant and washed three times. Magnetically separated beads were resuspended in 100 ul 2x SDS-sample buffer and boiled for 10 min at 95 °C to dissociate immunocomplexes from GFP-Trap MA beads. The beads were separated from the supernatant magnetically and the supernatant was subjected to immunoblotting.

### Cell transfection

NSC-34 cells were transfected with Lipofectamine 2000 (Invitrogen) according to the manufacturers’ protocol.

### NHEJ detection assay and nucleofection

NHEJ reporters, pimEJ5GFP or EJ2GFP-puro, were pre-digested with I-SceI restriction enzyme and the digestion products following purification were run on a 1% agarose gel to confirm that each reporter had been digested. Cells were co-transfected with mCherry-tagged TDP-43 wildtype, A315T, or Q331K, and pre-digested NHEJ reporter by nucleofection using Cell Line Nucleofector Kit V or Human Dermal Fibroblast Nucleofector™ Kit, according to the manufacturers protocol (Lonza). The efficiency of GFP restoration through NHEJ was estimated as the percentage of cells with both mCherry and GFP signals, in proportion to the total number of cells with mCherry expression [22, 38].

### TDP-43 mis-localisation assay

NSC-34 cells were cultured in DMEM media (Gibco) on 13 mm coverslips (Menzel-Glasser) in 24 well plates and transiently transfected with EGFP-tagged TDP-43 using Lipofectamine 2000 (Invitrogen). To induce DNA damage, cells were treated with 13.5 μM etoposide (E1383, Sigma) or DMSO as a control. After 48 h, cells were fixed with 4% PFA and mounted with Dako fluorescence mounting media. Images were taken with an epi-fluorescent microscope (Zeiss). For identification of SGs containing TDP-43, immunocytochemistry using a mouse anti-HuR (Invitrogen) antibody was performed. Primary cortical neurons were cultured in B27 neurobasal medium supplemented with 1% glutamate and 1% penicillin/streptomycin. Primary neurons were transfected with EGFP-tagged TDP-43 with Lipofectamine 2000 at 3 or 5 DIV. After 24 h neurons were treated with 13.5 μM (E1383, Sigma) or DMSO as a control for 1 h and fixed with 4% paraformaldehyde. Slides were mounted with Dako fluorescence mounting media. Images were taken with an epi-fluorescent microscope (Zeiss).

### Quantification of γH2AX, 53BP1 or DNA-PKcs foci

NSC-34 cells and primary neurons were selected for imaging only using the fluorescence/confocal microscopy channel displaying expression of GFP or mCherry-tagged TDP-43. Human fibroblasts were selected for imaging using the confocal microscopy channel displaying Hoechst staining of the nuclei. Hence, the channel displaying the presence of DNA damage foci was invisible to the researcher when the cells were selected for analysis. This strategy allowed us to identify transfected cells for analysis, but also it ensured that the researcher was blinded to the presence of DNA damage foci. Similarly, after imaging, cells were selected for analysis based only on the fluorescence microscopy channel displaying TDP-43 expression. Analysis of DNA damage foci was performed by three people. DNA damage images on immunofluorescently labelled NSC-34 cells or human fibroblasts were collected using a Zeiss LSM 880 confocal microscope with objective Plan-Apochromat 63x/1.4 Oil DIC M27 and z-stacks with intervals of 0.25 μm. Imaris software was used for three-dimensional reconstructions and quantification of the total area and volume of foci in NSC-34 cells. DNA damage analysis in primary cortical neurons or human fibroblasts was evaluated as the average number of γH2AX/53BP1 foci using ImageJ software. To quantify DNA damage in the TDP-43 rNLS mouse model, neurons specifically expressing the transgene were identified by double immunohistochemistry using an γH2AX antibody and an antibody specific for human, rather than mouse, TDP-43, because this model expresses human rNLS TDP-43 via a neuronal-specific promoter. Neurons were chosen at random within the cortex at different depths of coronal cross sections, and at similar levels in TDP-43 rNLS and control mice. Similar to the primary neurons, DNA damage was evaluated as the average number of γH2AX foci using ImageJ software.

### Super-resolution imaging and analysis

Fixed NSC-34 cells prepared as described above on coverglass were imaged using a custom home-built SR microscope as previously described [39, 40]. This system is based on an Olympus IX-81 frame equipped with 405 nm (Dragon, 50 mW), 488 nm (Toptica, 200 mW), 561 nm (Oxxius, 150 mW) and 638 nm (Oxxius, 150 mW) lasers. Excitation beams were combined using appropriate dichorics and focussed off a cube-mounted quad-band dichoroic filter (405/488/561/635 nm, Semrock) into an Olympus UPlanSApo oil-immersion 100X NA 1.49 TIRF objective. The excitation beam was laterally translated to achieve quasi-TIRF illumination manually optimized for each image depending on the nuclear imaging plane. Fluorescence emission was collected through the dichroic and a dedicated filter used to collect each colour channel sequentially (DAPI: 440/40 nm; Alexa Fluor 488: 525/50 nm; mCherry: 575lp with a 580/60 nm; Alexa Fluor 647: 700/75 nm (Semrock)). A further 1.6x magnification was engaged within the IX-81 to achieve an imaged pixel size of 98 nm on an Andor iXon Ultra 897 high-speed electron multiplying charge-coupled device (EMCCD) camera. 3000–5000 frames were captured at 10–50 Hz for each cell in each channel using the full 512 × 512 chip and a gain setting of 300. Colour channels were acquired sequentially in the order red-yellow-blue-violet. To generate optimal blinking, Alexa Fluor 488/647 samples were imaged in PBS containing 120 mM mercaptoethylamine (MEA). mCherry was transiently caged and uncaged in a buffer containing 80 mM MEA, 5% glucose, 400 μg/mL glucose oxidase, and 35 μg/mL catalase, similar to methods recently reported by others for SR of mCherry [41] and minimizing Alexa Fluor 647 photoconversion [42].

Output TIFF stacks were processed using the ImageJ plugin ThunderSTORM with default settings and then refined further as detailed by the plugin authors [43]. Specifically, we assigned a density filter (radius = 50 nm, minimum neighbours = 3), removed duplicates within the uncertainty limit, and merged multiple localizations within 20 nm and 1 frame. To correct for chromatic aberration, 20 nm rendered output images were registered using BunwarpJ to build channel-matched elastic transformation maps for localized images of multicolour Tetraspeck beads (Sigma). This elastic transformation was then applied to the SR images and the channels recombined in ImageJ to an RGB image. The Interaction Factor plugin was applied to each individual cell by manually drawing an appropriate ROI and automating an Otzu threshold [44]. Edge clusters were excluded and an average of 50 simulated images for each cell were used to determine the Interaction Factor. For display purposes within the manuscript, diffraction limited DAPI channels have been brightened and smoothed, and Gaussian blur and binary dilations have been applied to the SR images in order to make foci visible.

### Statistics

Data represent mean values and the standard error of the mean. Results were analysed using one-way or two-way analysis of variance (ANOVA) followed by Sidak, Tukey or Dunnett C post hoc tests, t-Student test or Mann Whitney test. The statistical analyses were performed using OriginPro 8.0, IBM SPSS or GraphPad Prism 7 software, and *p* value < 0.05 was considered statistically significant.

## Results

### Wildtype TDP-43 is protective against persistent DNA damage in neuronal cells

**Fig. 1:**
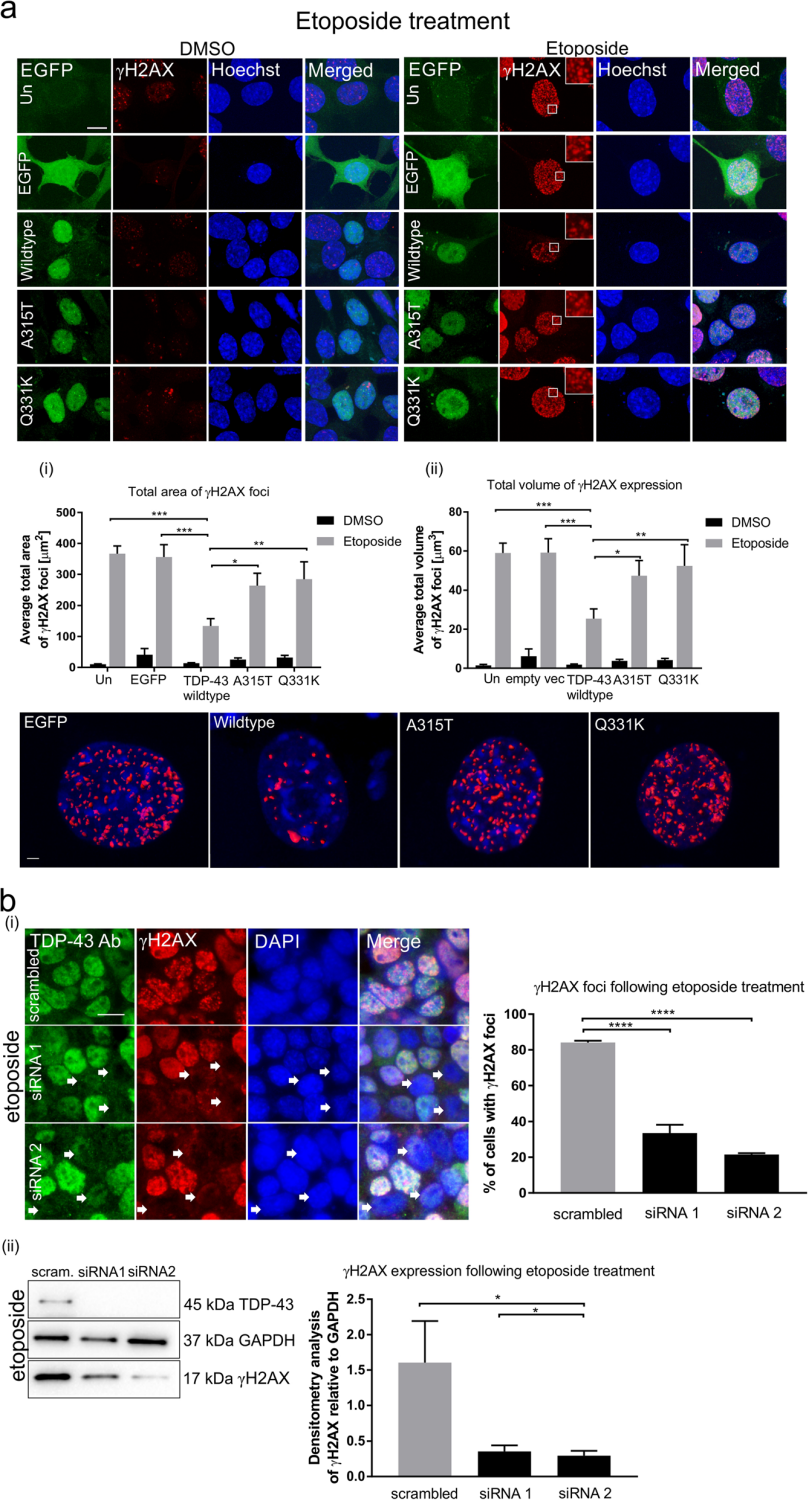

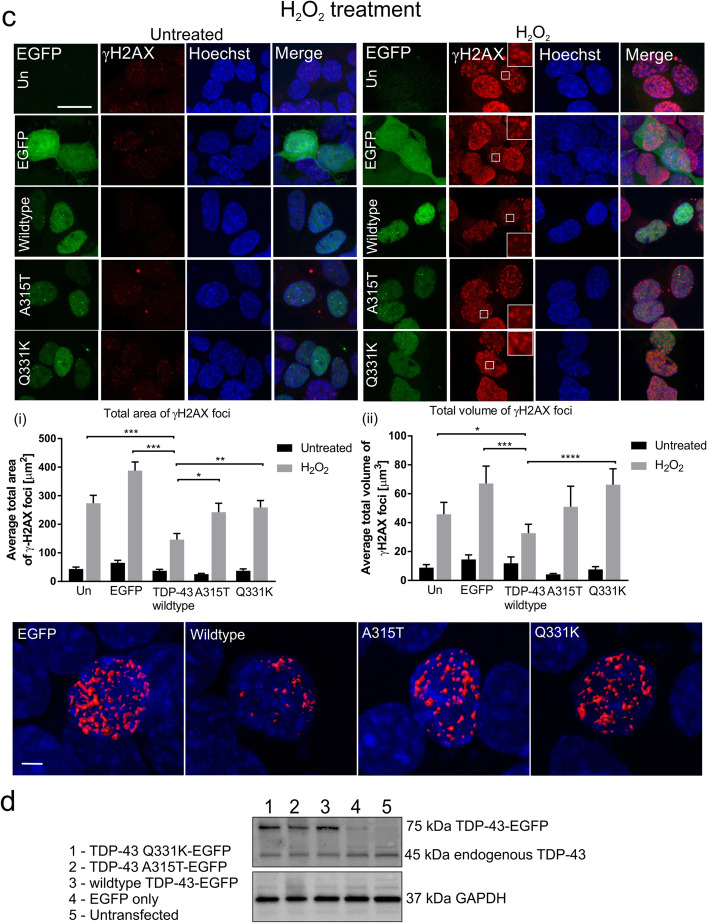
*Continued*

When DNA damage is induced and DSBs form, phosphorylation of the histone variant H2AX follows [45]. The resulting phosphorylated protein, γH2AX, rapidly forms foci that recruit DNA repair proteins, such as p53-binding protein 1 (53BP1). Hence the formation of γH2AX foci is a widely used, specific marker to assess DNA damage/repair [46]. Here, it was first examined whether TDP-43 functions in the DDR by examining the volume and area of γH2AX foci that form in neuronal cells using immunocytochemistry following induction of DNA damage. Two mechanisms were used to induce damage; either etoposide, a topoisomerase II inhibitor [47], or H_2_O_2,_ which induces DSBs by oxidative stress [48], a process that is widely implicated in neurodegeneration in ALS. Motor neuron-like NSC-34 cells were transfected with empty vector (encoding EGFP only) or either EGFP-tagged wildtype TDP-43, or ALS-associated mutants A315T or Q331K, and treated with either 13.5 μM etoposide or 100 μM H_2_O_2_ for 1 h, 24 h after transfection. The concentration of H_2_O_2_ was used as previously [48]. Following immunocytochemistry for γH2AX, Imaris software was used to assess the total area and volume of γH2AX foci using 3D reconstructions of confocal z-stack images. Quantitative analysis revealed that following either etoposide or H_2_O_2_ treatment, foci of significantly smaller area and volume were detected in cells expressing wildtype TDP-43 compared to untransfected cells (all ***p < 0.001) or those expressing EGFP only (area; both ***p < 0.001, volume; etoposide ***p < 0.001, H_2_O_2_ *p < 0.05), Fig. 1a (etoposide), and 1c (H_2_0_2_), or mutants (detailed below)). This result demonstrates that over-expression of wildtype TDP-43 is protective against persistent DNA damage induced by two different mechanisms. The DNA damage foci observed were all present in the nucleus, indicating that TDP-43 was protective against DNA damage in the nucleus, rather than in mitochondria.

### ALS-associated mutants A315T and Q331K TDP-43 proteins lose the protective function against DNA damage

A315T is a familial ALS-associated TDP-43 mutation and Q331K is a sporadic ALS-associated mutation [49]. Similar analysis of NSC-34 cells expressing EGFP-tagged mutants A315T or Q331K revealed that the γH2AX foci were of significantly larger area and volume in these cells compared to wildtype TDP-43 following etoposide (area: A315T, *p < 0.05, Q331K, **p < 0.01; volume: A315T, *p < 0.05, Q331K, **p < 0.01) or H_2_O_2_ treatment (area: A315T, *p < 0.05, Q331K, **p < 0.01; volume: Q331K ****p < 0.0001, Fig. 1a, c). Immunoblotting confirmed that the levels of transfected TDP-43 were similar amongst all cell populations, excluding the possibility that these results were caused by differences in transfection efficiency between the groups (Fig. 1d). No prominent differences in the size of the area/volume of γH2AX foci formed were evident between cells treated with etoposide and H_2_O_2_ for each group, revealing a broad protective effect for TDP-43, regardless of the mechanism of damage induction. These data therefore imply that ALS-associated mutants A315T and Q331K are not protective against the accumulation of DNA damage, implying that in ALS, TDP-43 loses its protective function against DNA damage.

**Fig. 2:**
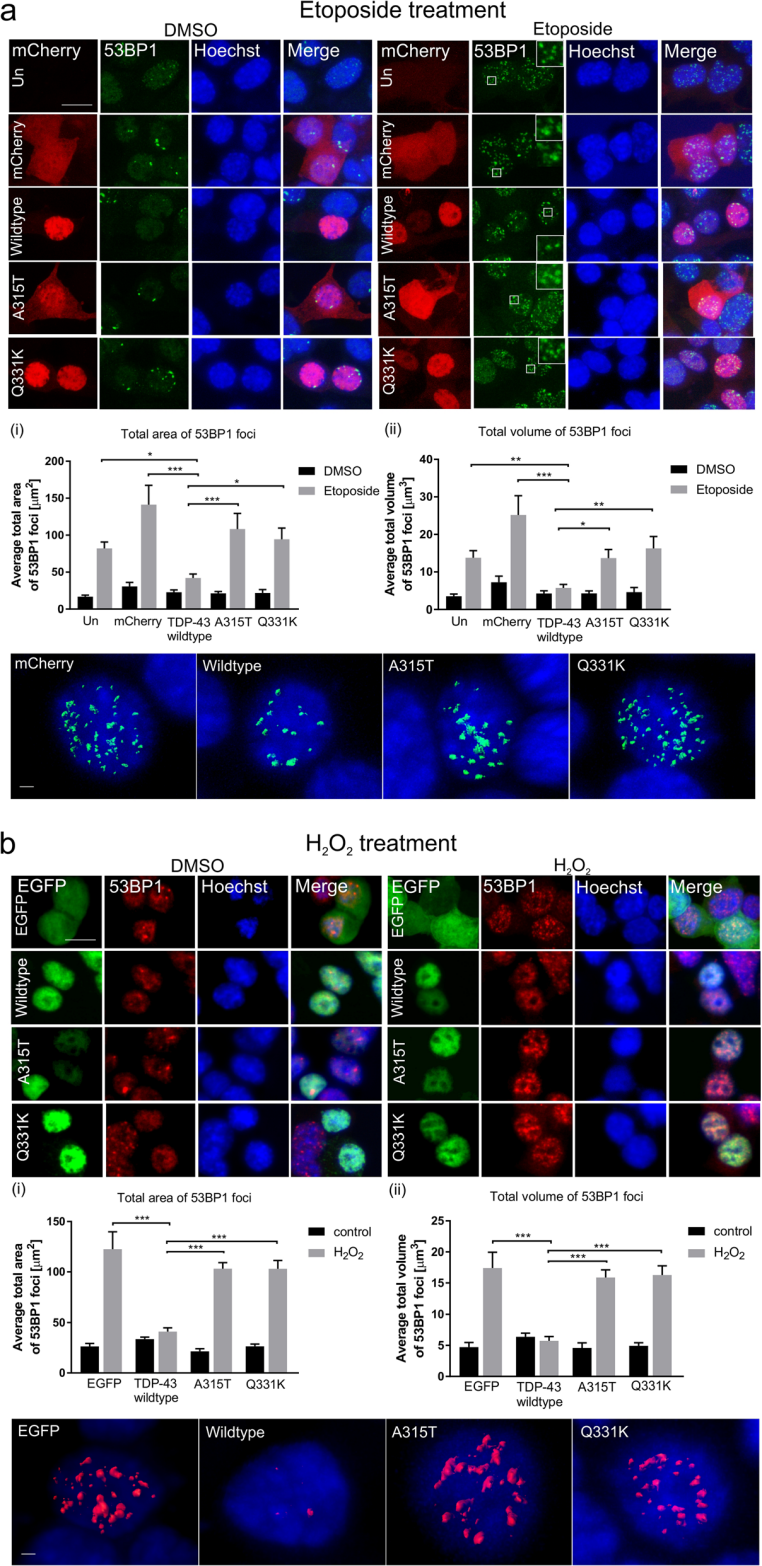
A315T and Q331K mutants do not prevent the formation of 53BP1 foci in NSC-34 cells *Top panels (****a****) and (****b****);* Confocal microscopy reveals that NSC34-cells expressing wildtype TDP-43 display less DNA damage compared to controls; untransfected (Un) or those expressing mCherry or EGFP only (mCherry, EGFP), determined by (i) total area, (ii) and total volume of 53BP1 foci after treatment with (**a**) 13.5 μM topoisomerase II inhibitor etoposide or (**b**) 100 μM H_2_O_2_ for 1 h. In contrast, cells expressing ALS-associated mutants A315T and Q331K are not protected from damage compared to wildtype TDP-43. Scale bar 10 μm. *Middle panels (****a****) and (****b****)*; Quantification was performed on 3D reconstructions of z-stack images using Imaris software. 2-way ANOVA with Sidak correction for multiple comparison. Mean ± SEM, *p < 0.05, **p<0.01, ***p < 0.001, three independent replicate experiments were performed (etoposide), two independent replicate experiments were performed (H_2_O_2_). At least 19 cells/group were analyzed. *Bottom panels (****a****) and (****b****);* Representative 3D reconstruction of confocal images of cells illustrating 53BP1 foci, Scale bar 2 μm

To confirm these findings, the formation of 53BP1 foci was next examined by immunocytochemistry. 53BP1 is an important regulator of the cellular response to DSBs. It is recruited early in the DDR, and it functions specifically in NHEJ, where it forms DNA damage foci [50]. We also examined cells expressing TDP-43 fused to another tag, mCherry, to eliminate the possibility that the results obtained were specific to EGFP-tagged TDP-43 only. Hence, in addition to the EGFP transfections as above, NSC-34 cells were also transfected with either mCherry-tagged wildtype TDP-43, mutants A315T or Q331K, or empty vector (encoding mCherry only). Cells were treated with etoposide or H_2_O_2_ as above. Using immunocytochemistry and confocal imaging, 53BP1 foci of significantly smaller area and volume were formed in cells expressing wildtype TDP-43 compared to untransfected cells (area: *p < 0.05, volume: **p < 0.01) or those expressing mCherry only (area: ***p < 0.001, volume ***p < 0.001, Fig. 2a) after etoposide treatment. Similarly, 53BP1 foci of significantly smaller area and volume were found in cells expressing wildtype TDP-43 compared to those expressing EGFP only (area: ***p < 0.001, volume ***p < 0.001, Fig. 2b) in H_2_O_2_ treated cells. This finding confirms that expression of wildtype TDP-43 is protective against DNA damage. It also implies that TDP-43 functions in NHEJ DNA repair. Similar analysis of cells expressing TDP-43 mutants revealed that the 53BP1 foci formed following etoposide or H_2_O_2_ treatment were of significantly larger area/volume in A315T cells (etoposide, area: ***p < 0.001, volume: *p < 0.05; H_2_O_2,_ area: ***p < 0.001, volume: ***p < 0.001) and Q331K cells (etoposide, area: *p < 0.05, volume: ** *p* < 0.01, Fig. 2a, H_2_O_2,_ area: ***p < 0.001, volume: ***p < 0.001, Fig. 2b) compared to wildtype TDP-43. Hence these data confirm that the ALS TDP-43 mutants do not possess the normal protective function of wildtype TDP-43 in DNA repair, likely leading to accumulation of DNA damage. Similar results were obtained in cells expressing EGFP-tagged TDP-43 proteins (Supplementary Fig. 1), thus confirming these results.

Next, we examined if TDP-43 knock down has the opposite effect to TDP-43 overexpression. We used two previously described specific siRNAs [35], targeting mouse TDP-43 or a non-targeting scrambled control sequence. Unexpectedly, in contrast to cells overexpressing TDP-43, TDP-43 knockdown prevented phosphorylation of H2AX following etoposide treatment, detected by immunostaining (****p < 0.0001, Fig. 1b**(i)**) and immunoblotting (*p < 0.05, Fig. 1b**(ii)**). Similar findings have been reported for FUS, which was shown to be involved in the recognition of DSBs by inhibiting H2AX phosphorylation [22]. Our data suggest that similar to FUS, TDP-43 may facilitate the phosphorylation of H2AX.

**Fig. 3:**
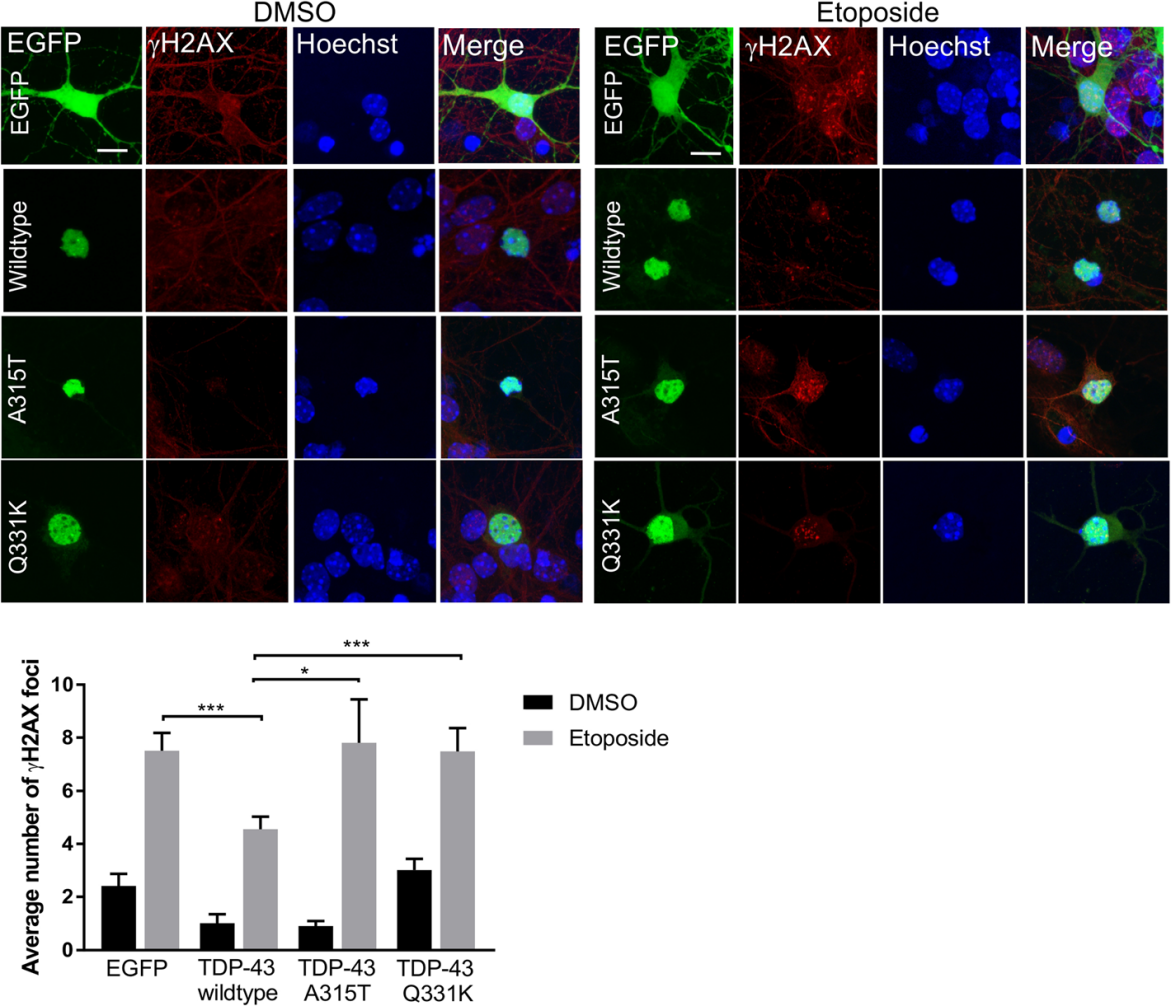
A315T and Q331K mutants do not prevent DNA damage in primary neurons. *Top/middle panels;* Confocal microscopy of mouse primary cortical neurons transfected with wildtype TDP-43, mutants A315T or Q331K, EGFP only (EGFP), or untransfected cells (Un), treated with 13.5 μM etoposide for 1 h. Immunocytochemistry was performed using anti-γH2AX antibodies. Scale bar 10 μm. *Bottom panel;* Quantification revealed less DNA damage γH2AX foci in neurons expressing wildtype TDP-43 after etoposide treatment compared to controls. In contrast, cells expressing ALS-associated mutants A315T and Q331K are not protected from damage compared to wildtype TDP-43. Two-way ANOVA with Tukey correction Mean ± SEM, *p  < 0.05, ***p < 0.001, *N* = 3, at least 20 cells/group were counted

To further corroborate these findings, primary mouse cortical neurons were transfected at DIV 3 with the EGFP tagged constructs as above, treated with etoposide, and immunocytochemistry and fluorescence microscopy were performed using antibodies against γH2AX. Here, the number of γH2AX foci formed was examined rather than the total area/volume, because unlike in NSC-34 cells where the foci are very dense and therefore difficult to differentiate between each other, in neurons they are discreet and easily distinguishable. Significantly fewer γH2AX foci were detected in primary neurons expressing wildtype TDP-43 after etoposide treatment, in comparison to those expressing EGFP only (***p < 0.001, Fig. 3). Similarly, significantly more γH2AX foci formed in primary neurons expressing either A315T (*p < 0.05) or Q331K (***p < 0.001) mutants compared to those expressing wildtype TDP-43 after etoposide treatment (Fig. 3). Together these findings therefore confirm that TDP-43 normally prevents persistent DNA damage, implying that it functions in DNA repair. However, this protective function is lost by the TDP-43 mutants, leading to the accumulation of DNA damage, thus implying that the integrity of the genome is compromised in ALS. Furthermore, because 53BP1 functions specifically in NHEJ, these findings imply that the process perturbed in ALS is loss of TDP-43 function in NHEJ.

### Wildtype TDP-43 is protective against DSBs formation, while ALS linked TDP-43 mutants lose this function

**Fig. 4:**
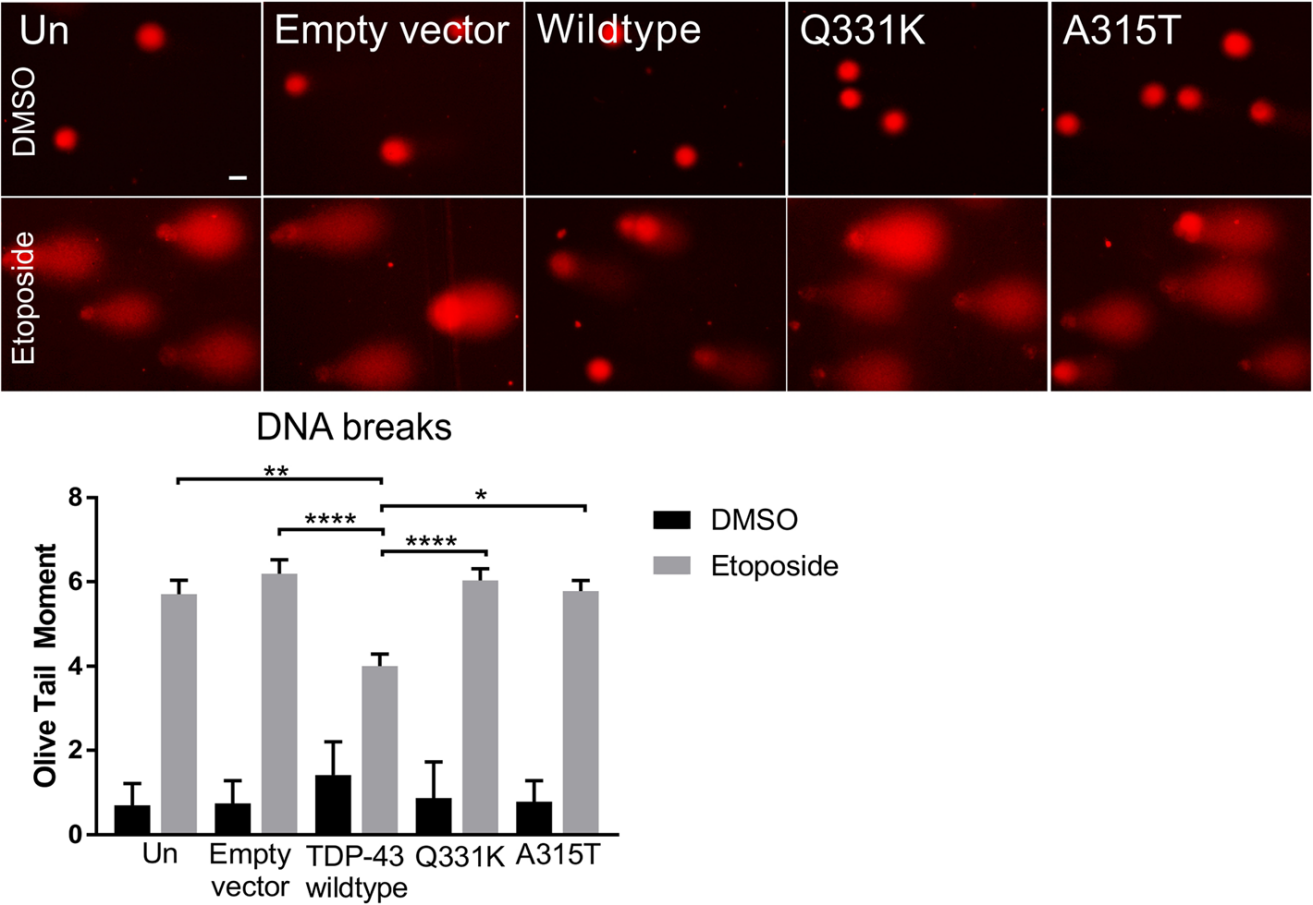
Wildtype TDP-43 prevents DSB accumulation in contrast to ALS-linked mutants A315T and Q331K. Neutral comet assay of Neuro2A cells expressing wildtype, Q331K, A315T or Empty vector (EGFP), or untransfected cells, only, treated with 13.5 μM etoposide for 1 h. **a** Representative images of comets. **b** Quantification of comets expressed as the Olive Tail Moment (OTM) revealed less DNA DSBs in cells expressing wildtype TDP-43 compared to Q331K, A315T mutants or EGFP and untransfected cells. Two-way ANOVA with Tukey correction Mean ± SEM, ****p < 0.0001, ** *p* < 0.01, * *p* < 0.05. N = 3, at least 50 cells/group were analysed

To determine if wildtype TDP-43 is protective against the formation of DNA DSBs, we performed a neutral comet assay on Neuro2A cells treated with etoposide as previous. Neuro2A cells were selected rather than NSC-34 cells because of their higher transfection efficiency. The ‘olive tail moment’ (OTM) of each comet is defined as the product of the comet tail length and the fraction of total DNA within the tail. Increased values indicate that more prominent DNA damage is present. This analysis revealed significantly lower OTM values for wildtype TDP-43 expressing cells after etoposide treatment compared to TDP-43 mutants A315T (*p < 0.05) and Q331K (****p < 0.0001). This indicates that fewer DSBs were present in wildtype TDP-43 cells, but not in mutant expressing cells, compared to control cells (****p < 0.0001 vs empty vector, *p < 0.01 vs Un, Fig. 4). These results are consistent with the findings obtained using immunocytochemistry against γH2AX and 53BP1 in NSC-34 cells.

### Wildtype TDP-43 is recruited to the sites of DNA damage, but this recruitment is perturbed by ALS-linked TDP-43 mutations

**Fig. 5:**
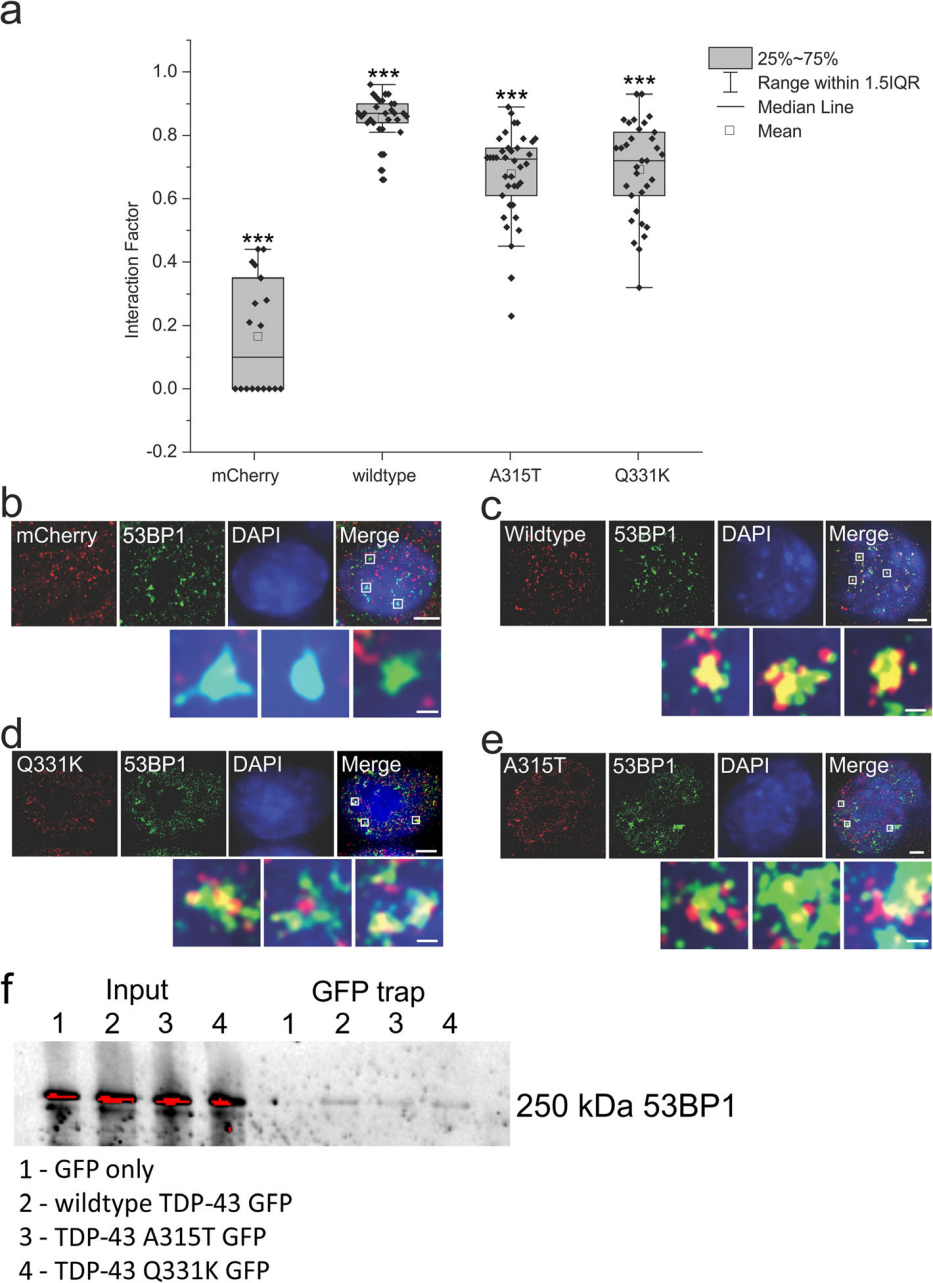
TDP-43 is recruited to 53BP1 DNA repair foci. Super-resolution imaging of NSC-34 cells expressing mCherry-tagged wildtype, Q331K, A315T TDP-43, or empty vector, enabled quantification of sub-diffraction colocalization between TDP-43 and 53BP1, in cells treated with 13.5 μM etoposide for 1 h. **a** Quantification of TDP-43/53BP1 co-localisation using the Interaction Factor ImageJ plugin, which was developed specifically for nuclear SR imaging. **b-e**. Representative SR images of etoposide-treated cells expressing mCherry-tagged wildtype, mutant Q331K, or A315T TDP-43, or empty vector, and immunostained for 53BP1. Low resolution epifluorescence DAPI images were acquired for nuclear localisation. Zoom-in regions of the boxed areas show prominent 53BP1 foci and mCherry-TDP-43 colocalization. t-test between wildtype compared to A315T, Q331K, and mCherry only, ***p < 0.001. Scale bars 3 μm in whole cell images, 250 nm in zoomed regions, *N* ≥ 30. (f) Immunoprecipitation of GFP Trap of NSC-34 cells expressing wildtype, Q331K, A331T TDP-43 or EGFP only. Wildtype and TDP-43 mutants co-immunoprecipitate with 53BP1, but not with control EGFP

These results taken together suggest that TDP-43 functions in DNA repair. However, it is unclear whether TDP-43 is recruited to DNA damage foci, where it could perform a specific DNA repair role at the DSB. Alternatively, it could perform a more indirect function in DNA repair, and therefore may not be recruited to sites of DNA damage. Hence, next we investigated the recruitment of TDP-43 to DNA damage (53BP1) foci in more detail by super-resolution microscopy (SR), using an assay previously developed for probing individual DSB sites [51]. In cells expressing mCherry-tagged TDP-43 proteins treated with etoposide as above, we combined mCherry-caging for single molecule localization microscopy [41] in order to visualize the distribution of TDP-43 with conventional *d*STORM SR imaging of immunolabelled 53BP1 [52]. We analysed the resulting two-colour SR images using the ‘Interaction Factor’ ImageJ plugin, an analytical tool developed specifically to assess multicolour SR images of dense distributions within the nucleus [44]. Wildtype TDP-43 strongly colocalized with 53BP1 foci, indicating a high level of recruitment and accumulation to DNA damage foci after DSB induction (Interaction Factor > 0.8, Fig. 5**a-e**). In contrast, mCherry consistently displayed a low Interaction Factor with 53BP1, indicative of low or random colocalization (Interaction Factor < 0.2, Fig. 5**a-e**). This confirmed that minimal colocalization artefacts were present due to crosstalk, bleed-through, or photoconversion, which we minimized using previously described methods [42]. Interestingly, despite loss of the DNA repair function of wildtype TDP-43, mutants A315T and Q331K were successfully recruited to DNA damage foci, albeit at statistically significant lower levels than wildtype TDP-43 (Interaction Factor > 0.5, A315T/Q331K ***p < 0.001, Fig. 5**a-e)**.

To confirm that TDP-43 is recruited to sites of DNA damage, we performed immunoprecipitation of lysates from NSC-34 cells expressing TDP-43 or controls expressing GFP only. We immunoprecipitated GFP-tagged TDP-43 or GFP lysates using ChemoTek GFP-Trap magnetic beads and performed immunoblotting for 53BP1. 53BP1 precipitated together with wildtype, A315T and Q331K TDP-43, but not with GFP only (Fig. 5f). The less recruitment to mutant TDP-43 to sites of DNA damage compared to wildtype TDP-43 may therefore contribute to the observed loss of DNA repair. However, it is probable that mutant TDP-43 also inherently possesses deficits in DNA repair functions, given that both mutants were recruited to sites of DNA damage and precipitated significantly more than GFP alone.

### Mutants TDP-43 impair classical NHEJ in ALS

**Fig. 6:**
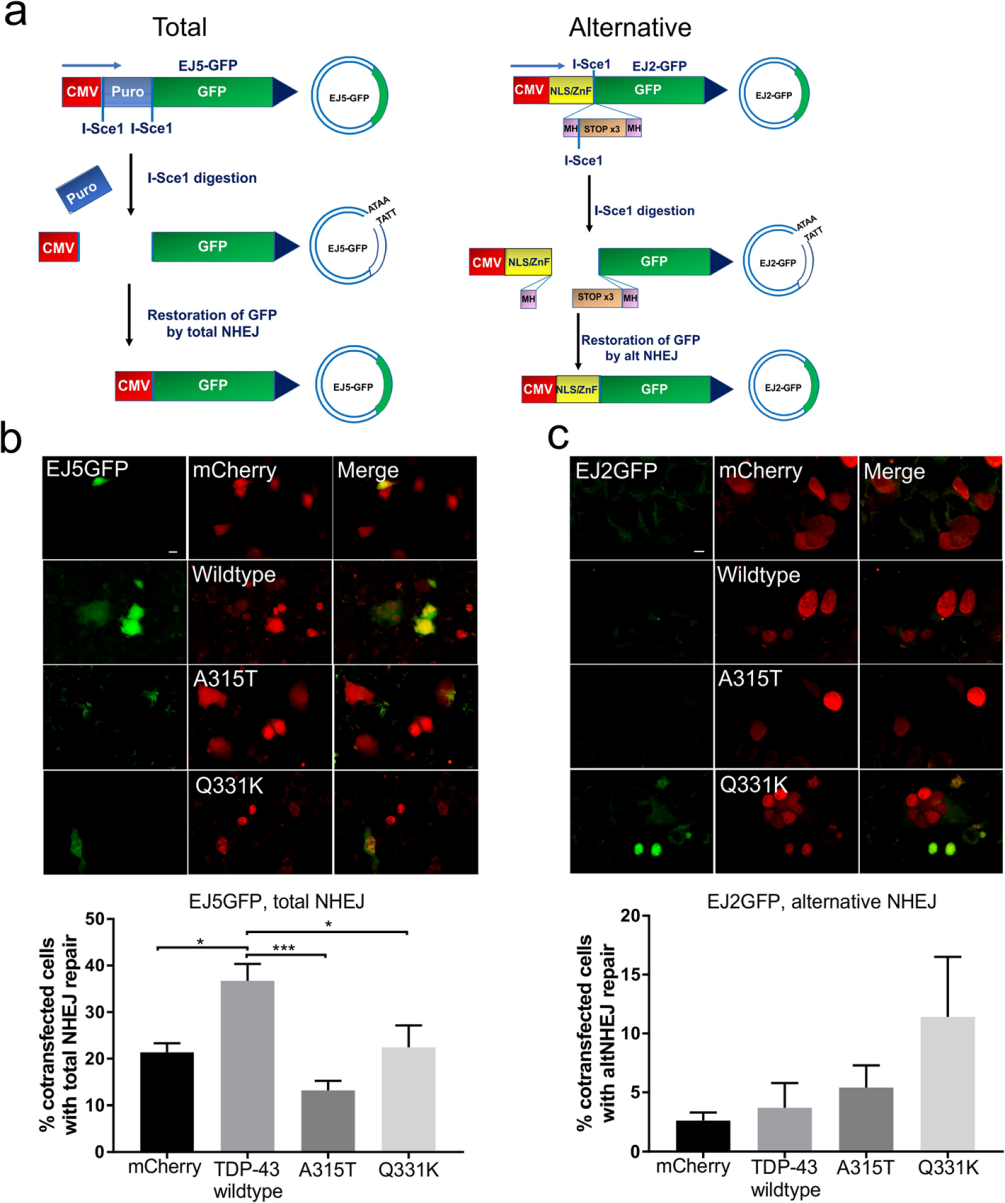
TDP-43 functions in classical NHEJ, whereas ALS-associated mutants lose this DNA repair function. **a** Schematic illustration of total NHEJ reporter, EJ5GFP (left panel), and alternative NHEJ, EJ2GFP (right panel) [38]. Successful repair of I-SceI digestion leads to restoration of GFP gene expression for each reporter. **b** Cells were co-transfected with digested NHEJ reporter EJ5GFP and either mCherry only (mCherry), or TDP-43 wildtype, or mutants A315T or Q331K, via nucleofection. The efficiency of total NHEJ (classical and alternative) was determined by the % of cells displaying GFP fluorescence. Quantification of GFP-positive cells in EJ5GFP transfectants indicated more total NHEJ in cells expressing wildtype TDP-43 compared to those expressing mutants or controls. One-way ANOVA, Mean ± SEM, *p < 0.05, ***p < 0.001. Scale bar 10 μm. At least 100 cells per group were counted, *N* = 6. (c) Cells were co-transfected with digested NHEJ reporter EJ2GFP and either mCherry only (mCherry), or TDP-43 wildtype, or mutants A315T or Q331K, via nucleofection. The efficiency of alternative NHEJ was determined by the % of cells displaying GFP fluorescence. Quantification of the number of GFP-positive cells in EJ2GFP transfectants indicates no significant differences in alternative NHEJ between cells expressing TDP-43 wildtype, mutants or controls, indicating that TDP-43 does not function in alternative NHEJ. One-way ANOVA, Mean ± SEM, *p* > 0.05. At least 57 cells per group were counted, N = 3

Next, to examine if wildtype TDP-43 facilitates NHEJ we performed assays for total NHEJ and alternative NHEJ using specific reporters, EJ5GFP and EJ2GFP respectively (Fig. 6a) [38]. EJ5-GFP contains a promoter separated from a GFP coding cassette by a *puro* gene, flanked by two I-SceI sites in the same orientation. Once the puro gene is excised by NHEJ repair of two I-SceI-induced DSBs, the promoter is joined to the expression cassette, leading to restoration of GFP, thus allowing detection of total NHEJ [38]. In contrast, the EJ2-GFP reporter contains an N-terminal tag (NLS/Zinc-finger) fused to GFP, where the intervening coding sequence is disrupted by an I-SceI site, followed by stop codons in all three reading frames, flanked by 8 nucleotides of microhomology. If annealed during alternative NHEJ, this restores the reading frame between the tag and GFP, resulting in deletion of 35 nucleotides [38]. This reporter is therefore specific for alternative NHEJ. We used NSC-34 cells for this purpose, because of their improved transfection efficiency compared to primary neurons [53]. Hence, EJ5GFP or EJ2GFP reporters were digested with I-SceI, and NSC-34 cells were co-transfected with mCherry-tagged TDP-43 constructs or mCherry empty vector, with pre-digested reporters. Total NHEJ was more efficient in cells expressing wildtype TDP-43 compared to cells expressing mCherry only, demonstrated by the presence of more cells with GFP expression, indicating restoration of EJ5GFP (*p < 0.05, Fig. 6b). Moreover, fewer cells expressing A315T (***p < 0.001) or Q331K mutants (*p < 0.05) were undergoing classical NHEJ compared to those expressing wildtype TDP-43, demonstrated by fewer GFP-positive cells (Fig. 6b). In contrast, expression of neither wildtype nor mutant TDP-43 significantly altered the proportion of GFP-positive cells when the alternative NHEJ reporter was used. Hence, neither mutant nor wildtype TDP-43 modulates alternative NHEJ (*p* > 0.05) (Fig. 6c). These data therefore imply that TDP-43 is required for classical, but not alternative NHEJ, thus confirming that TDP-43 facilitates DNA repair. However mutant TDP-43 lacks this DNA repair function.

### TDP-43 requires DNA-PK to mediate NHEJ repair

**Fig. 7:**
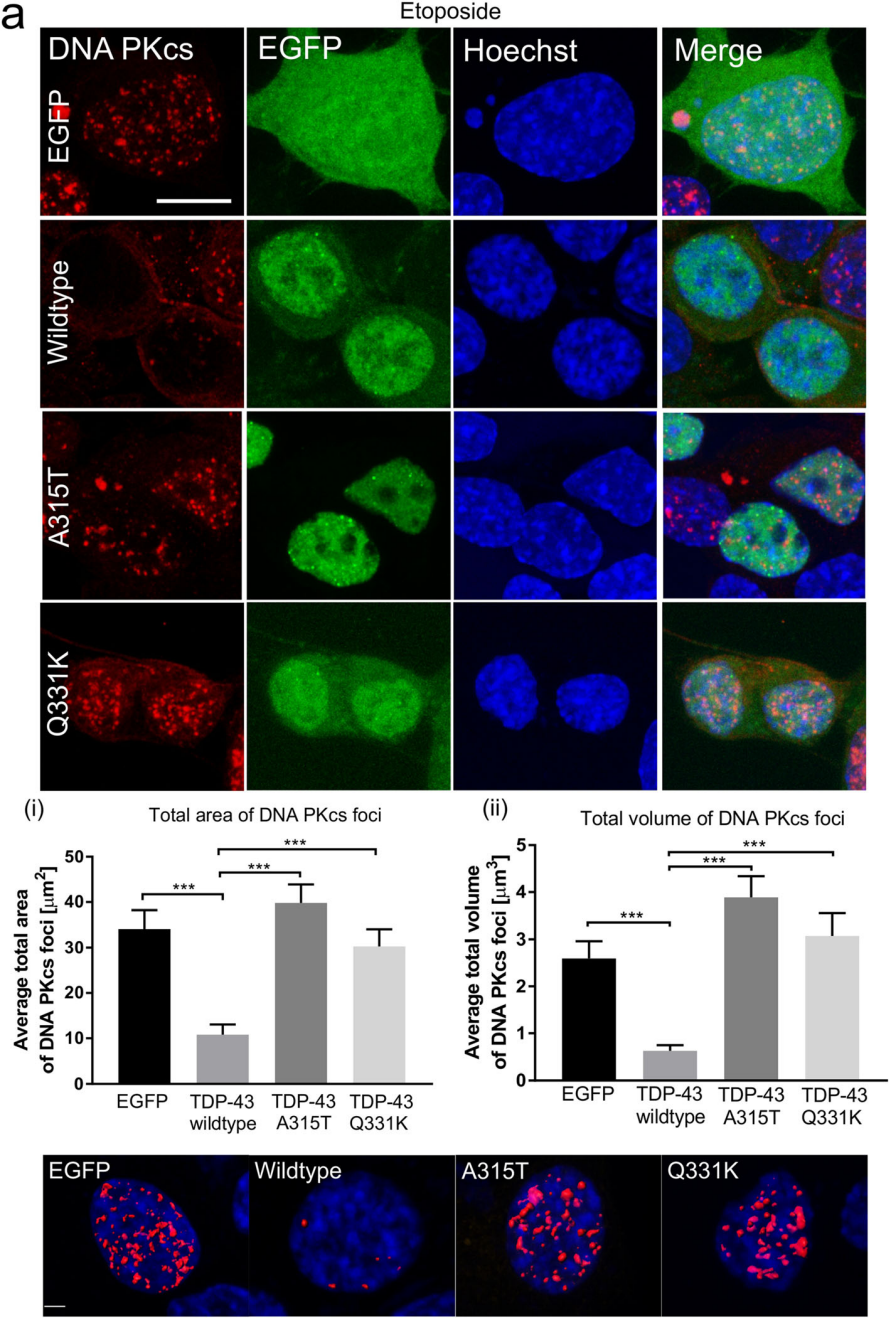

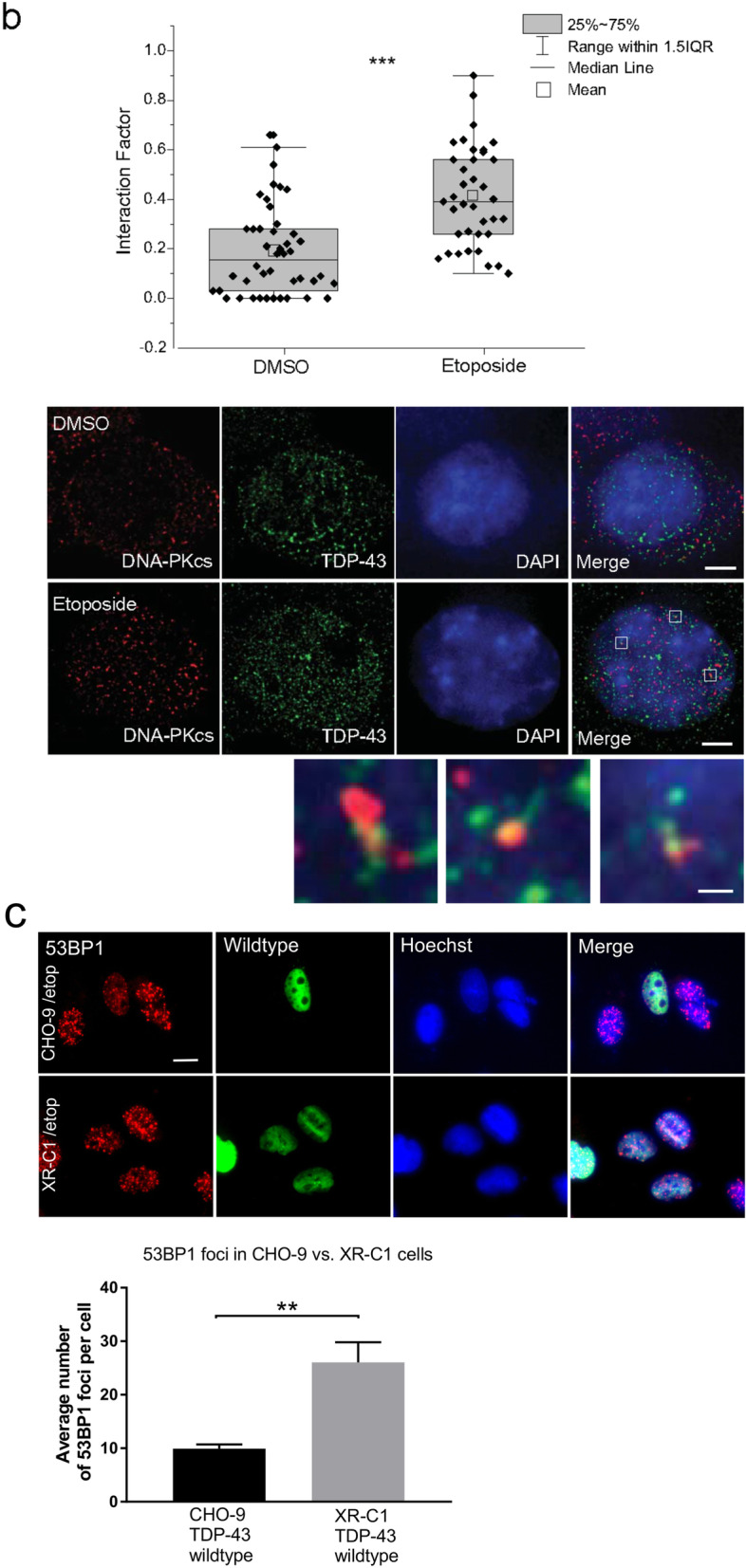
*Continued*

The DNA-dependent protein kinase (DNA-PK), consisting of Ku and DNA-PK catalytic subunits (DNA-PKcs), is a critical component that is recruited early in classical NHEJ [54]. To further probe the role of TDP-43 in NHEJ, we investigated the formation of DNA-PKcs foci using immunocytochemistry 1 h after etoposide treatment, in cells expressing wildtype, mutant TDP-43 or EGFP empty vector. These analyses revealed that the DNA-PKcs foci were significantly smaller in cells expressing wildtype TDP-43 in contrast to EGFP only (area and volume: ***p < 0.001, Fig. 7a). This result implies that less DNA damage remains following efficient repair by NHEJ because NHEJ is a fast process, that is normally completed within 30 min of the onset of DNA damage [50, 55]. Furthermore, the DNA-PKcs foci were significantly larger in both area and volume in cells expressing TDP-43 ALS-associated mutants A315T (***p < 0.001), or Q331K (***p < 0.001), compared to wildtype TDP-43 (Fig. 7a). Hence, mutant TDP-43 loses its NHEJ-specific DNA repair function in ALS. These findings therefore confirm that wildtype TDP-43 facilitates classical NHEJ, whereas ALS-associated mutants are defective in this property.

To confirm these findings, we next examined the recruitment of endogenous TDP-43 to DNA-PKcs foci in cells after etoposide treatment using SR microscopy as above. The Interaction Factor was calculated between TDP-43 and DNA-PKcs. Little colocalization was detected in DMSO treated cells, as expected (Interaction Factor < 0.2). However, this increased significantly upon induction of DNA damage by etoposide, indicating that TDP-43 is recruited to DNA-PKcs specifically when DNA damage is induced, and hence to regions of DSBs (Interaction Factor > 0.4). These data confirm that TDP-43 is recruited to sites of NHEJ repair (***p < 0.001, Fig. 7b).

Next, we examined the ability of wildtype and mutant TDP-43 to perform DNA repair in previously described DNA-PK deficient cells, XR-C1 [56]. XR-C1 and normal CHO-9 cells were transfected with wildtype or mutant TDP-43, or mCherry only constructs. DNA damage was induced with etoposide as previous and quantified as the average number of 53BP1 foci present. Wildtype TDP-43 retained its DNA repair ability in normal CHO-9 cells, but not in XR-C1 DNA-PK deficient cells, as indicated by fewer 53BP1 foci in these cells (**p < 0.01, Fig. 7c). Hence, this finding implies that TDP-43 is involved in DNA-PK dependent NHEJ repair. Moreover, expression of TDP-43 mutants A315T and Q331K was lethal for XR-C1 cells 24 h following transfection, implying an important role for proper DNA repair in the prevention of cell death in ALS (Table 1, Supplementary data).

### TDP-43 accumulation in the cytoplasm is associated with DNA damage in TDP-43 rNLS mice

**Fig. 8:**
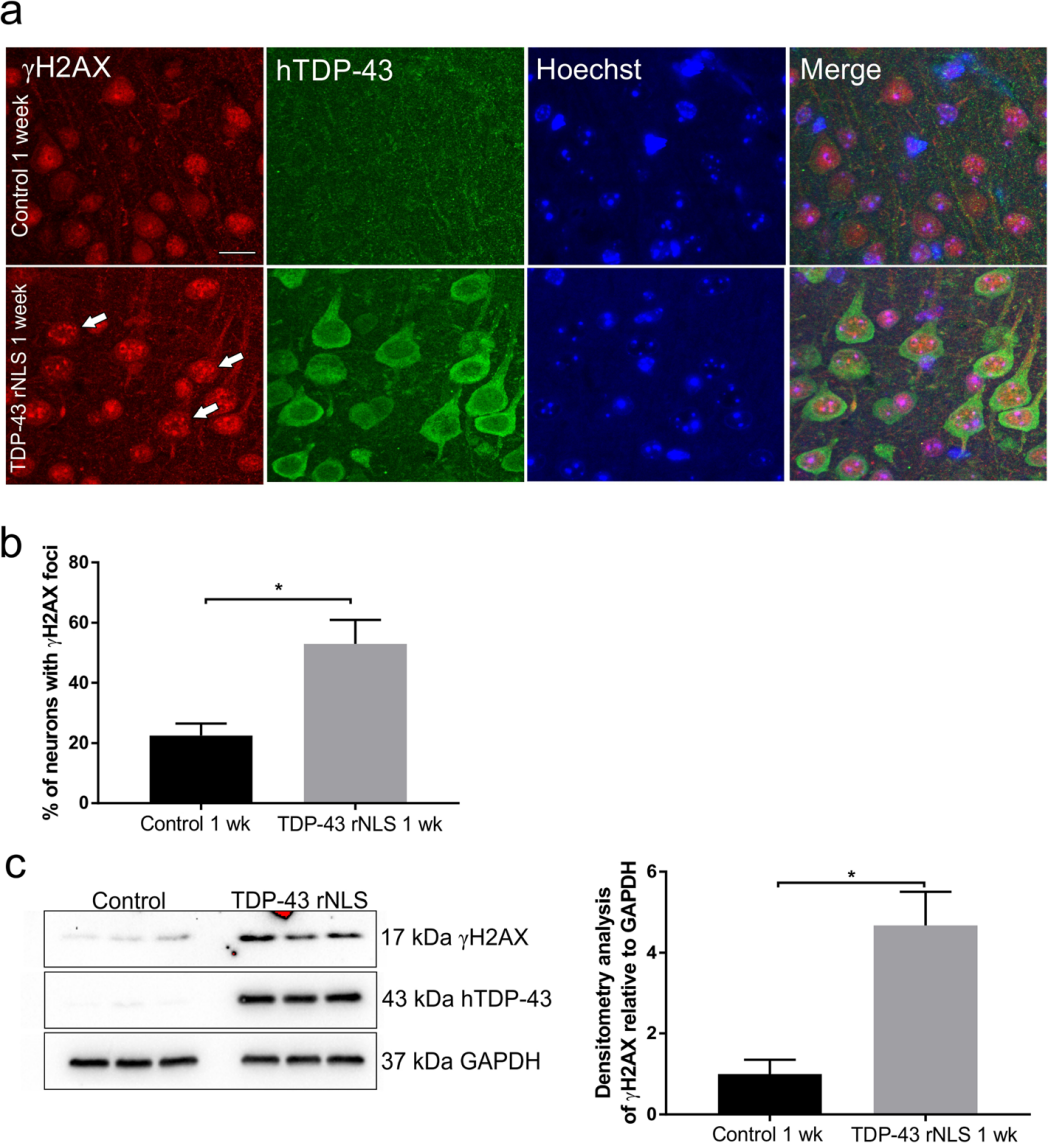
DNA damage correlates with TDP-43 pathology in a mouse model of ALS. DNA damage is evident in cortical neurons displaying cytoplasmic TDP-43 from rNLS mice at early stages of disease (1 week after doxycycline removal). Immunohistochemistry against (**a**) γH2AX, Scale bars 10 μM, white arrows indicate γH2AX foci (**b**) Quantification of images in (**a**) reveals significantly more cortical neurons with γH2AX foci in TDP-43 mice compared to non-transgenic aged and sex-matched controls, t-test, Mean ± SEM *p < 0.05. At least 180 neurons per mouse were counted, N = 3 (**c**) Immunoblotting reveals upregulation of γH2AX in cortical lysates from TDP-43 mice compared to controls, t-test *p < 0.05, Mean ± SEM, N = 3

Next, we examined the relationship between DNA damage and TDP-43 in a transgenic TDP-43 mouse model [12], which expresses cytoplasmic human TDP-43 by mutation of the nuclear localisation signal (NLS) under doxycycline (Dox)-suppressible control. Abundant cytoplasmic TDP-43 accumulates in the cortex of these animals 1 week after human TDP-43 expression begins, and they develop progressive neurodegeneration, motor deficits, and early death [12]. The accumulation is less pronounced in spinal, compared to cortical, motor neurons [12]. Hence, we next examined the cortex of these animals for DNA damage using Western blotting and immunohistochemistry, 1 week following Dox removal, which is prior to the onset of neuronal degeneration [12]. More γH2AX foci were present in cortical neurons of these animals (Fig. 8a and b, *p < 0.05) compared to controls, and elevated expression of γH2AX was also present in tissue lysates from these mice (Fig. 8c, *p < 0.05), revealing the accumulation of DNA damage. These data therefore indicate that DNA damage is already present, together with the onset of TDP-43 accumulation in the cytoplasm, in this mouse model of ALS. In addition, they reveal that DNA damage is triggered at early disease stages, implying it is relevant to pathophysiology and not simply a consequence of neurodegeneration. Furthermore, these findings suggest that DNA damage is associated with pathological localisation of TDP-43 in the cytoplasm, as well as familial ALS-associated mutant TDP-43, thus placing DNA damage onto the broad pathophysiology of ALS, not just cases caused by TDP-43 mutations.

### DNA damage induces features of TDP-43 pathology

**Fig. 9:**
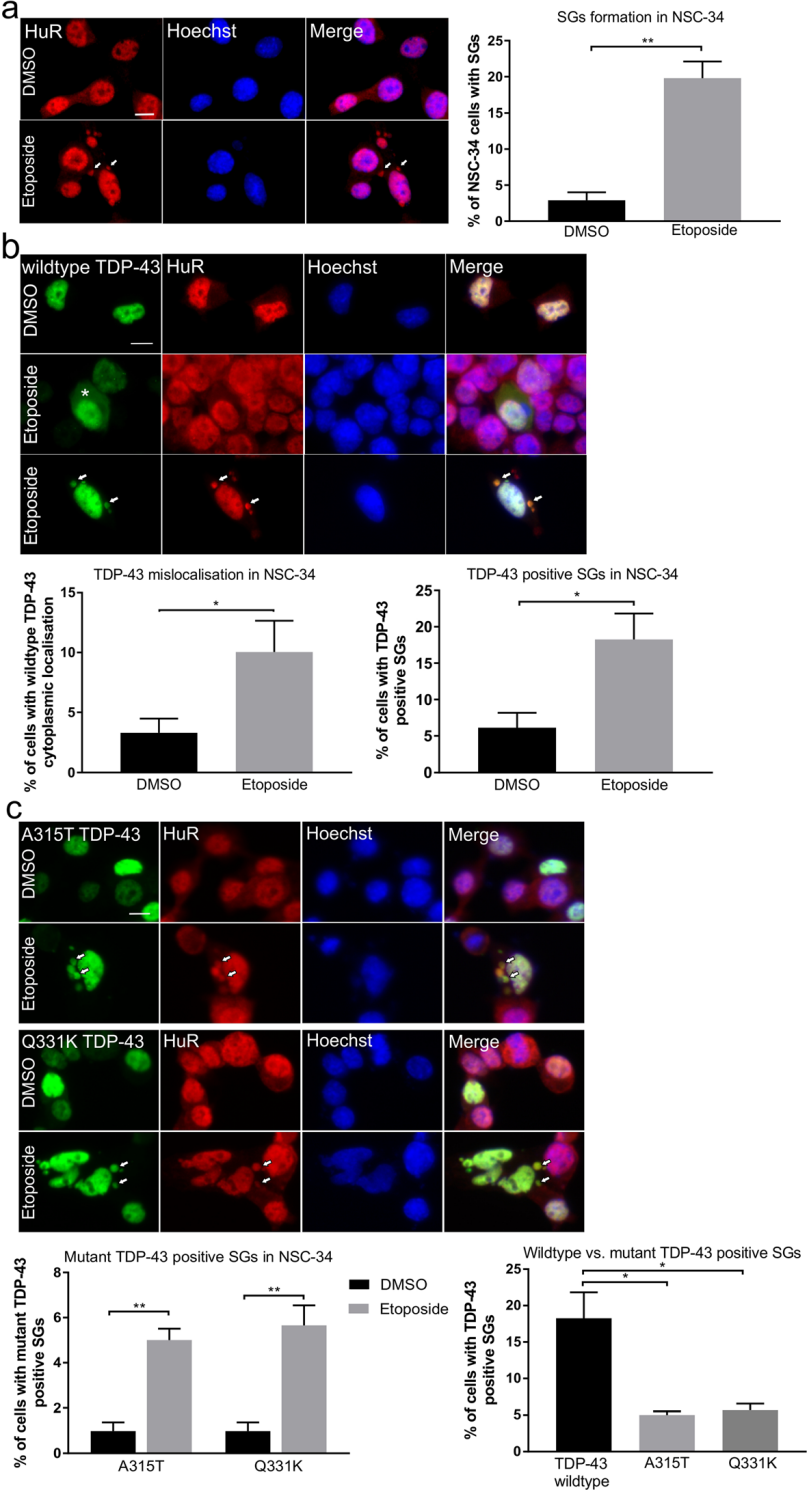

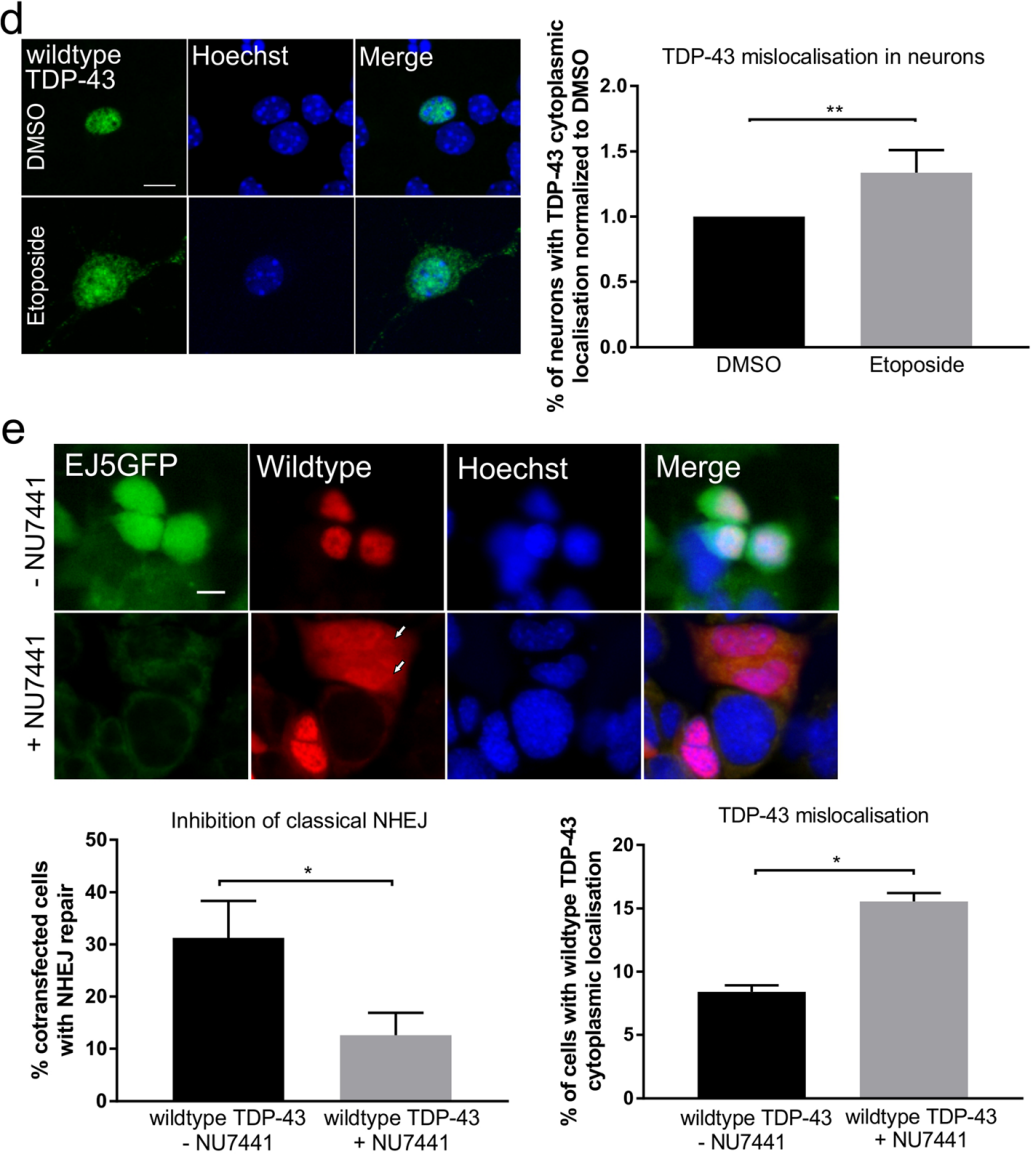
*Continued*

The presence of DNA damage in a TDP-43 mouse model and in cells expressing ALS-associated mutant TDP-43 implies that pathological forms of TDP-43 are associated with the DDR. Hence, we next examined whether induction of DNA damage directly induces features of TDP-43 pathology that are characteristic of ALS: mis-localisation of TDP-43 to the cytoplasm and stress granule (SG) formation. We examined this possibility in NSC-34 cells and primary mouse cortical neurons expressing wildtype or mutants Q331K or A315T TDP-43, treated with 13.5 μM etoposide for 48 h. In NSC-34 cells, induction of DNA damage by etoposide triggered the formation of SGs, confirmed by immunocytochemistry for HuR (**p < 0.01, Fig. 9a). Interestingly, more wildtype and mutant TDP-43 were also recruited to these SGs in cells treated with etoposide compared to controls, confirmed by immunocytochemistry for HuR **(**Fig. 9b and c, WT *p < 0.05, mutants **p  < 0.01). However, mutants TDP-43 Q331K and A315T were recruited to a lesser extent than wildtype TDP-43 **(**Fig. 9c, *p <  0.05). These results indicate that TDP-43 is recruited to SGs, which are implicated as precursors of inclusions in ALS [5, 6], following DNA damage. Moreover, mis-localisation of WTTDP-43 to the cytoplasm was significantly enhanced in NSC-34 cells treated with etoposide compared to controls treated with DMSO (*p < 0.05) **(**Fig. 9b). Similarly, we found enhanced mis-localisation of TDP-43 in mouse primary neurons after 1 h of etoposide treatment compared to DMSO treated neurons (**p < 0.01, Fig. 9d). SGs were not readily observed in primary neurons treated with etoposide, consistent with a recent study which demonstrated that SG assembly is impaired in cultured primary mouse cortical neurons and human neurons compared to cell lines [57]. These data therefore reveal that features of TDP-43 pathology are induced by DNA damage in ALS.

As our results implicate TDP-43 in NHEJ DNA repair and DNA damage in the formation of TDP-43 pathology, we next examined whether inhibition of classical NHEJ DNA repair can directly induce TDP-43 pathology. Therefore, we inhibited DNA-PK activity pharmacologically using selective DNA-PK inhibitor NU 7441 [58] in cells co-expressing the total NHEJ reporter EJ5GFP and wildtype TDP-43. NU 7441 significantly diminished GFP restoration, indicating less NHEJ, in cells treated with NU 7441 compared to untreated cells (*p < 0.05, Fig. 9e). Moreover, NHEJ inhibition with NU 7441 was accompanied by significantly more wildtype TDP-43 mis-localisation to the cytoplasm (*p < 0.05, Fig. 9e). This indicates that defective NHEJ DNA repair induces TDP-43 mis-localisation to the cytoplasm and in addition, it confirms that TDP-43 acts in classical NHEJ.

### Impaired NHEJ repair and features of TDP-43 pathology are present in human ALS

To confirm that our findings are related to human ALS, and are not an artefact of protein overexpression systems, we next investigated DNA damage, NHEJ repair and TDP-43 pathology in previously described fibroblasts obtained from subjects bearing the TDP-43 M337V mutation [33]. We obtained three different fibroblast lines, two from pre-symptomatic M337V cases and one from a M337V patient displaying symptoms of ALS, as well as three fibroblast lines from control individuals (Table 1).

**Fig. 10:**
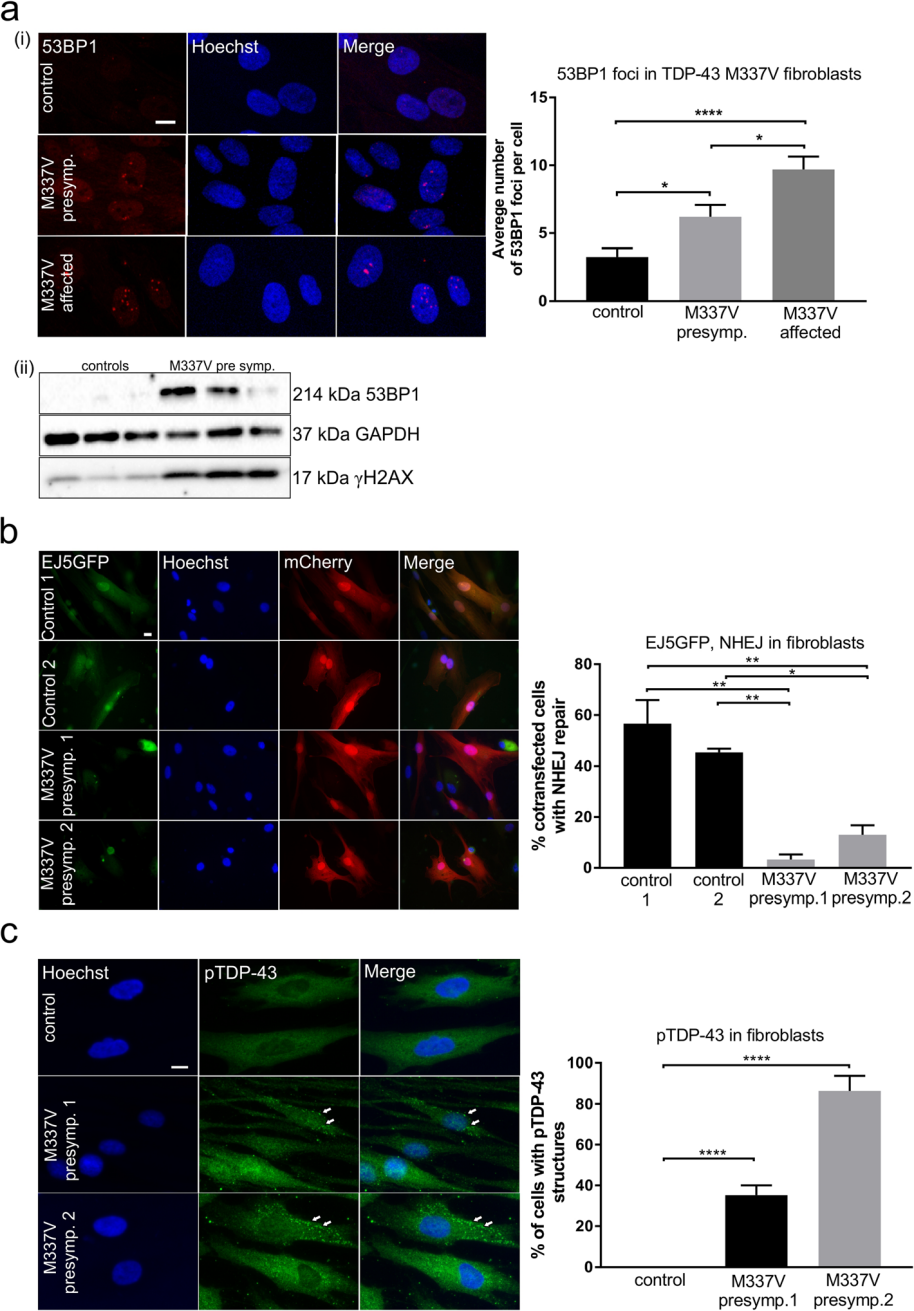
Impaired NHEJ is present in human ALS associated with TDP-43 M337V mutations. **a** *(top panel)* Immunocytochemistry for 53BP1 of TDP-43 M337V fibroblasts derived from ALS pre-symptomatic carriers, an affected ALS patient and control individuals. Confocal microscopy and quantification of the number of 53BP1 foci per cell revealed that significantly more DNA damage was present in ALS and pre-symptomatic fibroblasts compared to controls, and in the symptomatic compared to pre-symptomatic fibroblasts. One-way ANOVA with Tukey correction for multiple comparison. Mean ± SEM, ****p < 0.0001, *p < 0.05. 40 fibroblasts per individual were analyzed. Scale bar = 10 μm *(bottom panel)* Immunoblotting confirmed enhanced expression of DNA damage markers 53BP1 and γH2AX in TDP-43 M337V pre symptomatic compared to control fibroblasts (**b**) Fibroblasts from two TDP-43 M337V presymptomatic carriers and two control individuals were co-transfected with digested total NHEJ reporter EJ5GFP and empty mCherry vector via nucleofection. The efficiency of total NHEJ was determined by the % of cells displaying GFP fluorescence within mCherry transfected cells. Quantification of GFP-positive cells indicates significantly less NHEJ repair in TDP-43 M337V fibroblasts compared to controls. One-way ANOVA with Tukey correction for multiple comparison, Mean ± SEM, **p < 0.01, *p < 0.05. Scale bar 10 μm. At least 35 cells per group were analysed, N = 3. **c** TDP-43 M337V fibroblasts derived from pre-symptomatic carriers display pTDP-43 positive structures in the cytoplasm (white arrow), unlike control fibroblasts. One-way ANOVA with Tukey correction for multiple comparison, Mean ± SEM, ****p < 0.0001. Scale bar 10 μm. At least 82 fibroblasts were analysed within 10 fields per group

DNA damage was first examined by immunocytochemistry for 53BP1. The average number of 53BP1 foci per cell was significantly elevated in fibroblasts derived from the pre-symptomatic cases compared to the control lines (*p < 0.05, Fig. 10a**(i)**). Moreover, the number of 53BP1 foci/cell was significantly increased further in the affected ALS patient fibroblasts compared to those from control individuals (****p < 0.0001, Fig. 10a**(i)**) and the pre-symptomatic patient (*p < 0.05). These data therefore imply that DNA damage is present in mutant TDP-43-associated ALS and may increase during disease course. The fibroblasts obtained from the symptomatic M337V patients were difficult to culture because they were prone to undergo cell death. Hence the next experiments were performed on fibroblasts from pre-symptomatic cases only, which could be readily cultured. DNA damage was examined further by immunoblotting for DNA damage markers 53BP1 and γH2AX in the pre-symptomatic TDP-43 M337V fibroblasts, compared to control individuals (Fig. 10a**(ii) and (iii),** respectively). More expression of 53BP1 and γH2AX was present in TDP-43 M337V fibroblasts, compared to controls. These results therefore reveal the presence of DNA damage in human ALS fibroblasts with the TDP-43 M337V mutation.

Next, we investigated NHEJ repair in fibroblasts from pre-symptomatic TDP-43 M337V patients using the EJ5GFP reporter, to quantitate total NHEJ as above. TDP-43 M337V or control fibroblasts were co-transfected with pre-digested EJ5GFP reporter and empty mCherry vector as a control for transfection efficiency. NHEJ repair was found to be impaired in TDP-43 M337V fibroblasts compared to controls, demonstrated by fewer fibroblasts with GFP expression, indicating restoration of the EJ5GFP reporter (**p < 0.01, *p < 0.05, Fig. 10b). Finally, we investigated whether TDP-43 pathology is present in the fibroblasts displaying DNA damage, by immunocytochemistry using an antibody specific for phosphorylated TDP-43. In pre-symptomatic TDP-43 M337V fibroblasts, phosphorylated TDP-43 positive structures, reminiscent of aggregates, were present in 37% and 89% of cells. However, these aggregates were not detected in control fibroblasts (Fig. 10c, ****p < 0.0001). These results therefore imply that there is an association between TDP-43 pathology and DNA damage in cells without artificial protein overexpression. These data together confirm the relationship between impaired DNA repair and human ALS associated with TDP-43 mutations.

## Discussion

Maintaining the integrity of the genome is of vital importance to all cells, but particularly neurons, which are highly susceptible to DNA damage due to their inability to divide and undergo HR, one of the two major mechanisms for repairing DSBs. Furthermore, it is well established that the accumulation of unrepaired DNA lesions triggers apoptosis and leads to neurodegeneration [59]. In this study we demonstrate that TDP-43 facilitates repair of DSBs, the most cytotoxic type of DNA damage, in the nucleus via NHEJ. To investigate TDP-43 pathology, the A315T and Q331K mutants were chosen because they represent TDP-43 pathology found in either sporadic (Q331K) or familial (A315T) ALS. Thus, by including mutations found in sporadic and genetic forms of disease, ALS can be considered more generically. It is also known that the TDP-43 Q331K mutation affects the normal function of TDP-43 in RNA splicing in mice models [60]. In contrast to wildtype TDP-43, TDP-43 ALS mutants Q331K and A315T lose this important function, leading to the accumulation of DNA damage.

Our study therefore implicates failure of DNA repair mechanisms by TDP-43 in the pathophysiology of ALS, implying that the integrity of the genome is compromised in this disorder. There is controversy as to whether loss of TDP-43 nuclear function or gain of cytoplasmic toxic functions (or both) are most relevant to neurodegeneration in ALS. Our findings point to a loss of nuclear function of TDP-43 as a pathogenic process in ALS. Whilst the RNA-related functions of TDP-43 are well described, this study also demonstrates that an important DNA-specific function is perturbed in ALS. In addition, induction of DNA damage leads to mis-localisation of TDP-43 to the cytoplasm, and the formation and recruitment of TDP-43 to SGs, revealing that DNA damage can induce features characteristic of TDP-43 pathology. SGs positive for mutant TDP-43, Q331K and A315T, were also formed upon induction of DNA damage. Interestingly, however fewer SGs were formed in these cells compared to those expressing wildtype TDP-43. The results are consistent with the notion that functional TDP-43 is required for efficient SG dynamics [57].

Similarly, inhibition of NHEJ leads to TDP-43 mis-localisation to the cytoplasm. Therefore, this raises the possibility that persistent DNA damage and defective DNA repair could further perturb the nuclear functions of TDP-43 in DNA repair, thus exacerbating nuclear DNA damage. Surprisingly, TDP-43 knockdown prevented H2AX phosphorylation, an important event in the detection of DSBs, suggesting that TDP-43 functions in the recognition of DSBs. Similar findings were reported for FUS, implying that it functions in the detection of DSBs [22].

The formation of 53BP1 foci in cells expressing TDP-43 implicates NHEJ, the only DSB repair mechanism available to neurons, as the mechanism of DNA repair mediated by TDP-43, and that which is lost from mutant TDP-43 in ALS. NHEJ is already more error-prone than HR [61], and when this mechanism fails, cells use an even more error-prone method - alternative NHEJ - which is mediated by low fidelity DNA-polymerase θ [62, 63]. Specific reporter assays for these two pathways confirmed that TDP-43 functions in total, but not alternative NHEJ, revealing a role for TDP-43 in classical NHEJ and loss of this function in ALS. In contrast, wildtype TDP-43 does not function in alternative NHEJ. Therefore, we identify a role for TDP-43 in classical but not alternative NHEJ, revealing that TDP-43 functions in the DSB repair mechanism most important to neurons. Importantly, this function is impaired by mutant TDP-43 in ALS.

To obtain more detailed insights into TDP-43 function in NHEJ, we examined the formation of DNA-PKcs foci in NSC-34 cells following DNA damage. Classical NHEJ is initiated when a Ku70/80 heterodimer binds to DSBs, which recruits DNA-PKcs to create the DNA-PK complex, forming DNA damage foci. This event is therefore early in classical NHEJ and it facilitates binding of the other DNA repair proteins involved in this process [54, 64]. We found significantly fewer DNA-PKcs foci in cells expressing wildtype TDP-43 in contrast to those expressing TDP-43 ALS-mutants (Q331K or A315T). This suggests that more effective DNA repair is present in cells expressing wildtype TDP-43 compared to those expressing TDP-43 ALS mutants, because NHEJ occurs rapidly and is complete by 30 mins after the onset of damage [50, 55]. Moreover, we found that wildtype TDP-43 lost its repair ability in cells lacking DNA-PK, whereas mutant TDP-43 triggered the death of these cells (Fig. 7c, Supplementary Table 1). Furthermore, SR imaging and co-immunoprecipitation revealed specific recruitment of TDP-43 to both 53BP1 and DNA-PK-immunopositive foci after DNA damage (Fig. 5**,** Fig. 7b). This result contrasts with untreated cells, revealing that TDP-43 functions directly at the DSB site alongside other proteins involved in classical NHEJ, thus implying that it has a specific function in DNA repair. These findings are consistent with a previous study which demonstrated that TDP-43 localizes at sites of transcription-associated DNA damage, and colocalises with γH2AX in undamaged cells [30]. Also, our findings are consistent with a more recent study in which TDP-43 was recruited to DSB sites in neuroblastoma cells treated with bleomycin, where a role in NHEJ repair was implicated [31]. Conditional depletion of TDP-43 induced DSBs by impairing NHEJ repair [31], similar to the effect of expression of TDP-43 ALS linked mutants, A315T and Q331K in this study. However, Mitra et al. 2019 based their research mostly on TDP-43 depletion. Although they demonstrated a correlation between DNA damage and TDP-43 nuclear clearance in post-mortem spinal cord ALS tissue, they did not demonstrate a link between impaired NHEJ and TDP-43 ALS-associated pathology.

In this study we also demonstrate that TDP-43 is involved in the phosphorylation of γH2AX, further implying that TDP-43 functions upstream of 53BP1. In addition, DNA damage was detected in mutant TDP-43 Q331K patient tissues, and in vitro, TDP-43 Q331K mis-localisation to the cytoplasm prevented nuclear translocation of the XRCC4-DNA Ligase 4 complex [32]. In this study we now demonstrate that expression of TDP-43 Q331K and other TDP-43 ALS mutants, M337V and A315Ts impair NHEJ repair. We show loss of the normal TDP-43 classical NHEJ DNA repair by mutants Q331K and A315T, independent of TDP-43 mis-localisation to the cytoplasm, in cells treated with both etoposide and H_2_O_2_, a source of oxidative DNA damage. Moreover, we also demonstrate that impaired NHEJ was present in fibroblasts bearing the M337V TDP-43 mutation.

Further SR imaging demonstrated that the TDP-43 mutants are recruited less efficiently to DNA damage foci than wildtype TDP-43, implying that this is one mechanism triggering impairment of NHEJ in ALS. However, it is important to note that the TDP-43 mutants were recruited more than mCherry only to DNA damage foci, revealing that at least a proportion of mutant TDP-43 does reach the sites of DNA damage. It is therefore possible that additional deficits also exist for mutant TDP-43, such as functional deficiencies in the repair of DSBs, which combined with impaired recruitment of TDP-43 to these sites, enhances the lack of DNA repair and thus the accumulation of DNA damage. Together, these results therefore reveal that TDP-43 is involved in DNA-PKcs-dependent DDR, confirming its role in NHEJ. Importantly, the TDP-43 mutants (Q331K and A315T) were defective in this process, revealing that they lose this important and specific cellular function, resulting in accumulation of DNA damage in ALS.

Our study also describes a compelling link between DNA damage, defects in DNA repair and TDP-43 pathology in ALS. The presence of pathological forms of TDP-43 in affected neurons is a characteristic feature of 97% of ALS [4] and pathological inclusions colocalize with SG markers in ALS and FTD in human tissue and cellular models [65, 66]. A prevalent hypothesis is that inclusion formation in ALS and FTD is driven by the failure of SGs to disassemble and phase separation of low complexity domains of RNA binding proteins [67, 68]. Consistent with this notion, SG assembly disrupts nucleocytoplasmic transport and contributes to neurodegeneration [69]. However, there was previously no evidence linking DNA damage and defects in DNA repair to TDP-43 pathology. Here we show for the first time that induction of DNA damage results in mis-localisation of TDP-43 to the cytoplasm, where it becomes recruited to HuR-positive SGs. Importantly, DNA damage also directly induces the formation of SGs and inhibition of NHEJ induces TDP-43 mis-localisation. These findings therefore directly link key pathological features of ALS to DNA damage and deficits in TDP-43 function in NHEJ. They also imply that DNA damage and repair is important in the pathophysiology of ALS associated with TDP-43.

Consistent with this notion, DNA damage was present at early stages of disease in TDP-43 rNLS mice, coincident with TDP-43 accumulation in the cytoplasm and prior to the presence of neurodegeneration in this mouse model. Importantly, TDP-43 rNLS mice express mis-localised wildtype TDP-43 rather than mutant TDP-43, and therefore arguably this model is closer to sporadic ALS than TDP-43-associated familial ALS. Since these mice show an early decrease in levels of normal endogenous TDP-43 in the nucleus of neurons due to auto-regulation of expression [12], the increase in DNA damage in these animals implies that an early loss of the normal nuclear protective function of TDP-43 occurs in disease. These results therefore place DNA damage onto the pathophysiology of sporadic disease, rather than just the rare genetic forms. Indeed, DNA damage has been previously detected in motor neurons of sporadic ALS patients [70]. Wildtype TDP-43 is present in almost all ALS patients, but unlike in the normal population, in ALS it is mis-localised to the cytoplasm and becomes aggregated and aberrantly modified. Hence in sporadic ALS, it is possible that depletion of TDP-43 from the nucleus may result in an inability to perform its normal DNA repair function, which results in an accumulation of DNA damage in patient motor neurons. This may result from environmental stressors in sporadic disease.

Increasingly DNA damage is becoming linked to neurodegenerative diseases [71]. Apoptosis in spinal motor neurons follows DNA damage [72] and DNA repair enzymes are up-regulated in ALS brains, indicating increased DNA damage. Similarly, in ALS mouse models, motor neuron degeneration was associated with DNA damage [73]. TDP-43 shares similarities with FUS, which also functions in DNA repair, whereas ALS-associated FUS mutants lose this function [74–76]. The TDP-43 knockdown studies suggest that TDP-43, similar to FUS, functions in the recognition of DSBs by H2AX phosphorylation. The results from our study therefore highlight further similarities between these two proteins. We and others also previously demonstrated that DNA damage is induced by C9ORF72 dipeptide repeat proteins (DPRs) and is present in C9ORF72-ALS patient motor neurons [28]. Importantly, expression of the C9ORF72 DPRs is closely associated with, and known to precede, TDP-43 pathology in cellular models [77]. The results of the current study therefore raise the possibility that expression of the C9ORF72 repeat expansion in ALS triggers DNA damage, which subsequently induces TDP-43 pathology in ALS, suggesting a potential mechanistic link between the C9ORF72 mutation and TDP-43 pathology.

Finally, we investigated the relevance of our findings to human ALS using fibroblasts from an ALS patient bearing the TDP-43 M337V mutation and two pre-symptomatic TDP-43 M337V carriers. Enhanced DNA damage was present in affected and pre-symptomatic fibroblasts. Importantly, significantly more DNA damage was present in pre-symptomatic affected fibroblasts compared to presymptomatic fibroblasts, implying an active role for DNA damage in the development of ALS. However, it is possible that the presence of the TDP-43 M337V mutation renders pre-symptomatic fibroblasts more vulnerable to DNA damage in vitro. Similarly, total NHEJ was impaired in TDP-43 M337V pre-symptomatic fibroblasts compared to control fibroblasts. The upregulation of 53BP1, which promotes classical NHEJ [78], is also consistent with our findings of impaired classical NHEJ. Moreover, following the presence of DNA damage, aggregate-like structures positive for phosphorylated TDP-43 were evident, reminiscent of TDP-43 pathology. These results therefore confirm the relationship between DNA damage and defects in DNA repair in TDP-43 mutant-associated ALS. They also confirm that our findings are relevant to human ALS, and the presence of DNA damage in cells expressing physiological levels of TDP-43 confirms that our results are not an artefact of artificial protein overexpression systems.

**Fig. 11:**
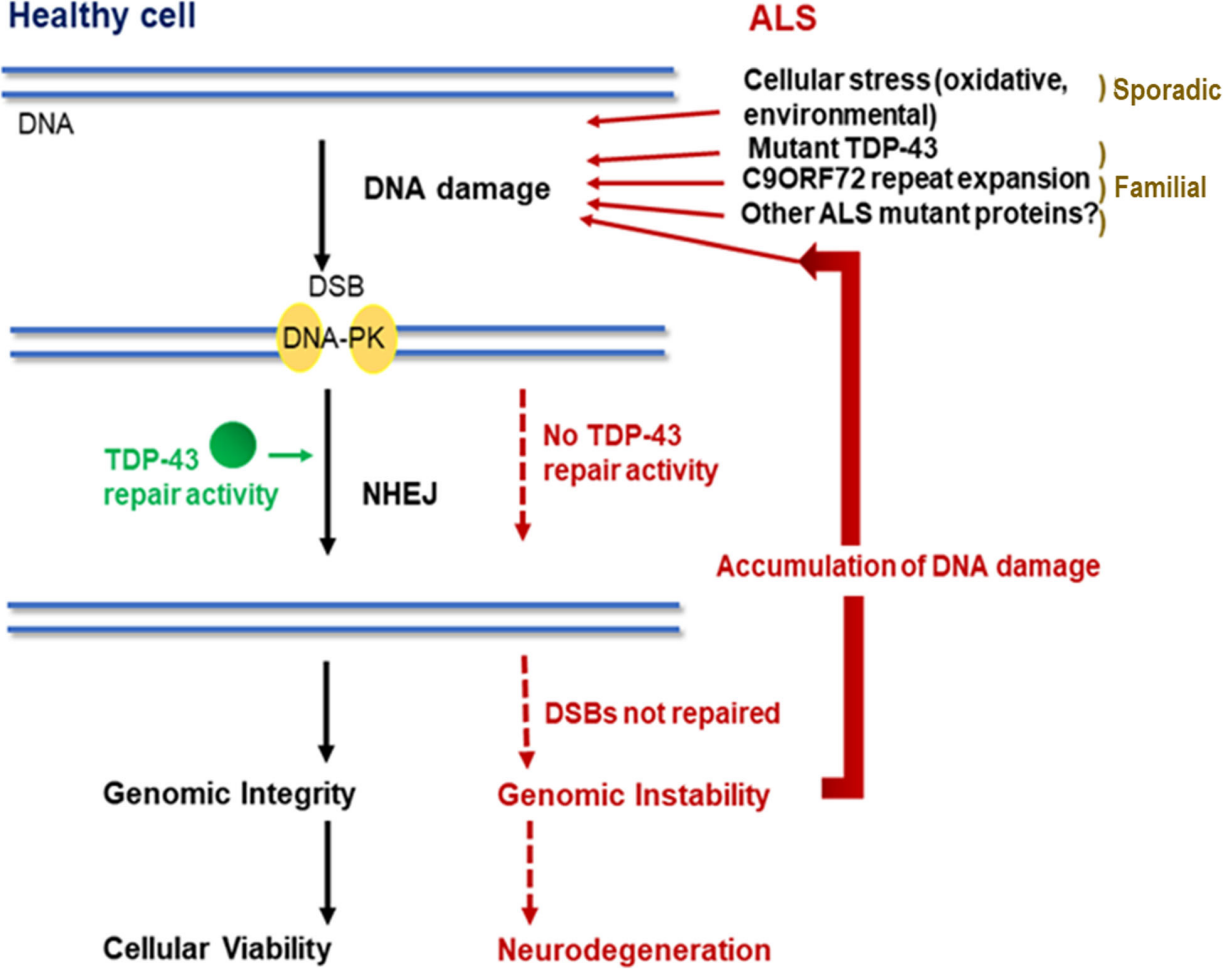
Hypothetical model illustrating the relationship between DNA damage and neurodegeneration in ALS. TDP-43 normally functions in NHEJ to repair cytotoxic DSBs, the only DNA DSB repair mechanism available in post-mitotic neurons. However, in familial ALS, misfolded mutant TDP-43 lacks this function, leading to DNA damage and neurodegeneration. Similarly, other mutant proteins associated with ALS also induce DNA damage. In sporadic ALS, oxidative stress or other forms of cellular stress induce DNA damage. In all cases, this results in TDP-43 mis-localisation to the cytoplasm, loss of nuclear TDP-43 and impairment of NHEJ. The resulting DNA damage induces further TDP-43 mis-localization to cytoplasm and more perturbation of DNA repair in the nucleus, creating a viscous cycle which exacerbates nuclear DNA damage, inducing TDP-43 pathology, and leading to genomic instability and eventually neurodegeneration

The findings of our study raise the possibility that failure of DNA repair processes by mutant TDP-43 may trigger a vicious cycle, in which the resulting accumulation of DNA damage triggers TDP-43 mis-localisation in the cytoplasm, inducing more loss of nuclear TDP-43. This would enhance existing DNA damage and perturb DNA repair even further, leading to significant accumulation of DNA damage during the normal human lifespan, triggering neurodegeneration. Figure 11 illustrates a hypothetical scheme based on the findings of this study, which explores the relationship between DNA damage and pathological forms of TDP-43. Similarly, DNA damage was recently shown to enhance the cytoplasmic mis-localisation of FUS [76]. Together these studies and our results imply that the detrimental relationship between DNA damage and pathological forms of misfolded proteins could be a common theme in ALS [76]. This study also identifies inhibition of DNA damage and/or enhancement of DNA repair as possible novel therapeutic strategies in ALS.

## Conclusions

In this study we demonstrate that TDP-43 mediates DNA repair via the NHEJ pathway. In contrast, TDP-43 ALS mutants Q331K and A315T lose this function, contributing to the accumulation of DNA damage. Our findings also reveal a link between DNA damage and the formation of pathological forms of TDP-43 in ALS. Whilst the RNA-related functions of TDP-43 are well described, this study identifies an important DNA-specific function that is perturbed in ALS.

## Supplementary information

**Additional file 1: Supplementary Figure 1.** A315T and Q331K mutants do not prevent the formation of 53BP1 foci (EGFP-tagged constructs). *Top panels;* Confocal microscopy of NSC34-cells expressing wildtype TDP-43 display less DNA damage compared to controls; untransfected (Un) or EGFP only cells (EGFP), determined by (i) total area, (ii) and total volume of 53BP1 foci after treatment with 13.5 μM topoisomerase II inhibitor etoposide. In contrast, cells expressing ALS-associated mutants A315T and Q331K are not protected from damage compared to wildtype TDP-43. Scale bar 10 μm. *Middle panels*: Quantification was performed on 3D reconstructions of z-stack images using Imaris software. 2-way ANOVA with Sidak correction for multiple comparison. Mean ± SEM, *p < 0.05, **p < 0.01, ***p < 0.001. At least 20 cells/group were analyzed. *Bottom panels;* Representative 3D reconstruction of confocal images of cells illustrating 53BP1 foci, Scale bar 5 μm.

**Additional file 2: Supplementary Table 1.** Q331K and A315T TDP-43 mutants cause cell death in cells with DNA-PK deficiency. Expression of Q331K and A315T TDP-43 mutants in XR-C1 cells lacking DNA PK was lethal in all three replicates (crosses). Wildtype CHO-1 cells with Q331K and A315T expression survived in 2 out of 3 replicates (check marks).

## Data Availability

The datasets used and/or analysed during the current study are available from the corresponding author on reasonable request.

## Abbreviations

TDP-43: TAR DNA-binding protein 43
ALS: Amyotrophic lateral sclerosis
NHEJ: Non homologous end joining
DNA-PK: DNA-dependent serine/threonine protein kinase
FTD: Frontotemporal dementia
hnRNP: Heterogenous nuclear ribonucleoprotein
SGs: Stress granules
DDR: DNA damage response
DSBs: Double-strand breaks
DNA-PKcs: Catalytic subunit of DNA-dependent protein kinase
SSBs: Single stranded breaks
SETX: Senataxin
VCP: Valosin-containing protein
NEK1: NIMA Related Kinase 1
C21orf2: Chromosome 21 Open Reading Frame 2
CCNF: G2/mitotic-specific cyclin-F
53BP1: p53-binding protein 1
FUS: Fused in sarcoma
DIV: Days in vitro
OTM: Olive tail moment
SR: Super resolution
