# Supplementary figures and images for: Impaired NHEJ repair in amyotrophic lateral sclerosis is associated with TDP-43 mutations
Source: Mol Neurodegener. 2020 Sep 9;15:51. doi: 10.1186/s13024-020-00386-4 (PMC7488163; doi:10.1186/s13024-020-00386-4)

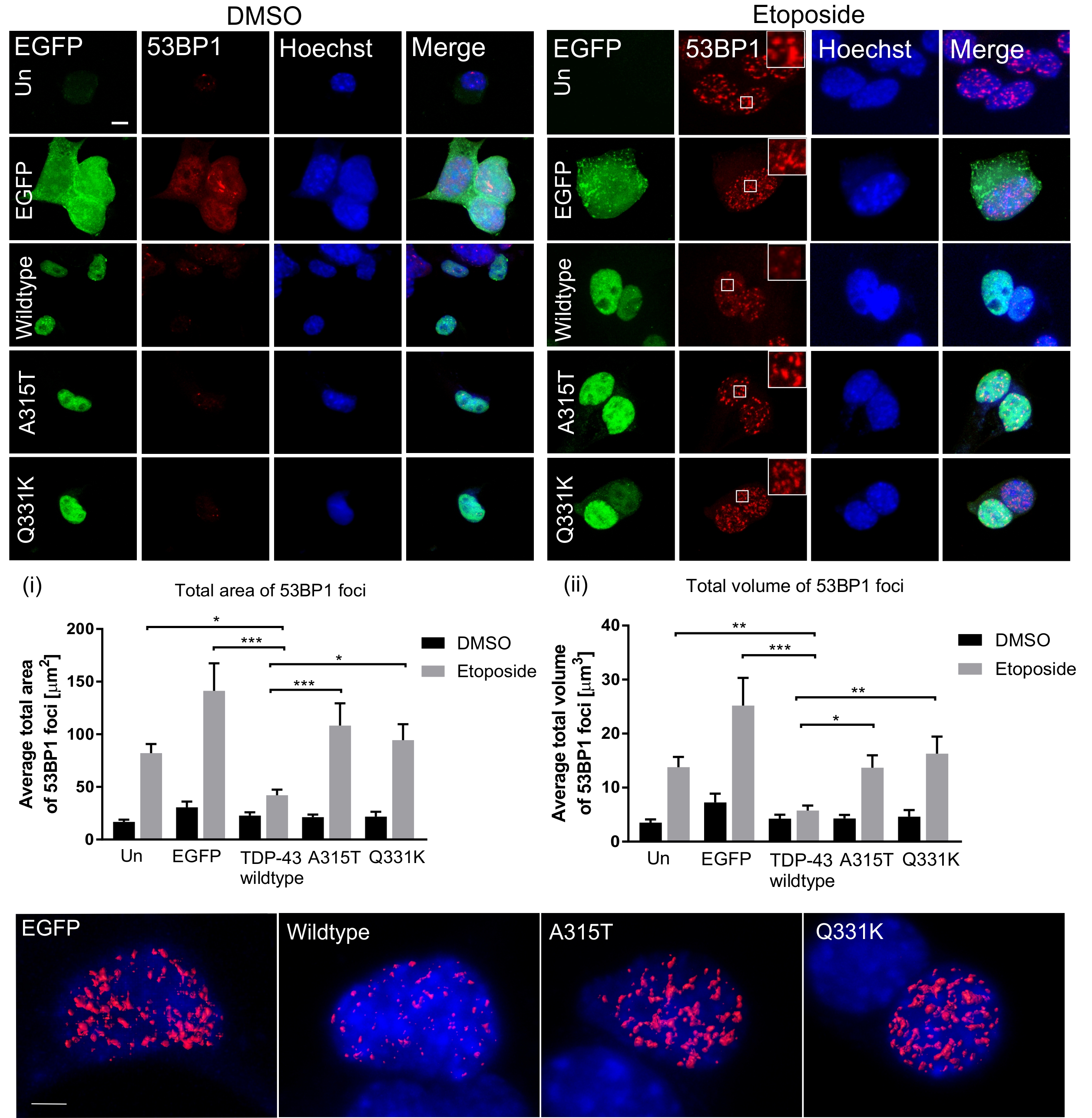

Supplement: Supplementary file 1 — Additional file 1: Supplementary Figure 1. A315T and Q331K mutants do not prevent the formation of 53BP1 foci (EGFP-tagged constructs). Top panels; Confocal microscopy of NSC34-cells expressing wildtype TDP-43 display less DNA damage compared to controls; untransfected (Un) or EGFP only cells (EGFP), determined by (i) total area, (ii) and total volume of 53BP1 foci after treatment with 13.5 μM topoisomerase II inhibitor etoposide. In contrast, cells expressing ALS-associated mutants A315T and Q331K are not protected from damage compared to wildtype TDP-43. Scale bar 10 μm. Middle panels: Quantification was performed on 3D reconstructions of z-stack images using Imaris software. 2-way ANOVA with Sidak correction for multiple comparison. Mean ± SEM, *p < 0.05, **p < 0.01, ***p < 0.001. At least 20 cells/group were analyzed. Bottom panels; Representative 3D reconstruction of confocal images of cells illustrating 53BP1 foci, Scale bar 5 μm. [file 13024_2020_386_MOESM1_ESM.jpg]

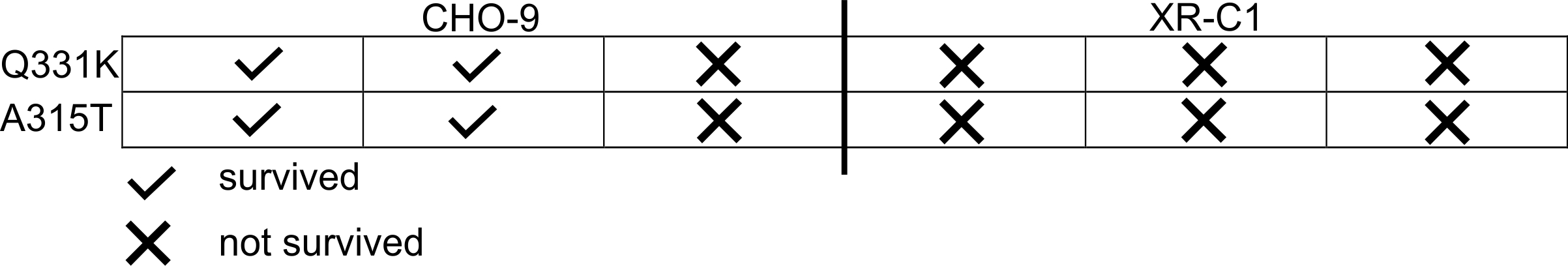

Supplement: Supplementary file 2 — Additional file 2: Supplementary Table 1. Q331K and A315T TDP-43 mutants cause cell death in cells with DNA-PK deficiency. Expression of Q331K and A315T TDP-43 mutants in XR-C1 cells lacking DNA PK was lethal in all three replicates (crosses). Wildtype CHO-1 cells with Q331K and A315T expression survived in 2 out of 3 replicates (check marks). [file 13024_2020_386_MOESM2_ESM.jpg]
